# Receptor-induced activation of MERS-CoV spike revealed by time-course cryo-EM

**DOI:** 10.1371/journal.ppat.1014545

**Published:** 2026-10-08

**Authors:** Yajie Wang, Xiyuan Pan, Yuxuan Jiang, Zhimin Liu, Yinong Qiu, Zhenguo Chen, Lei Sun

**Affiliations:** Shanghai Institute of Infectious Disease and Biosecurity, Shanghai Fifth People’s Hospital, Institutes of Biomedical Sciences, Shanghai Medical College, Fudan University, Shanghai, China; Institut de Biologia Molecular de Barcelona IBMB-CSIC, SPAIN

## Abstract

The Middle East respiratory syndrome coronavirus (MERS-CoV) initiates infection by engaging its host receptor dipeptidyl peptidase 4 (DPP4), which activates the viral spike (S) glycoprotein. However, how receptor binding is structurally coupled to membrane fusion has remained unclear. Here, using cryo–electron microscopy, we determined structures of MERS-CoV spike-DPP4 complexes at defined incubation times, capturing a series of conformational states spanning prefusion, receptor-bound, and postfusion conformations. These structures reveal that receptor-binding domain (RBD) opening progressively weakens S1-S2 interactions, while DPP4 binding may induce conformational drift and loosening of the S2 fusion core. These structural changes might destabilize spike trimers, promote S1 dissociation, and prime the fusion machinery. Compared with the SARS-CoV-2 spike, the MERS-CoV spike exhibits greater conformational plasticity, consistent with a lower threshold for receptor-induced fusion. In addition, the postfusion S2 structure suggests that the HR2 upstream linker may function as a conformational switch during six-helix bundle assembly. Together, these findings outline a structural pathway. for receptor-induced activation of the MERS-CoV spike and provide a structural framework for the development of antiviral strategies targeting dynamic fusion intermediates.

## Author summary

Middle East respiratory syndrome coronavirus (MERS-CoV) enters host cells using its spike, which binds the cellular receptor DPP4 and subsequently drives membrane fusion. Although this process is essential for viral infection, the structural changes linking receptor binding to membrane fusion have remained poorly understood. In this study, using cryo-electron microscopy, we captured a series of receptor-bound conformational states together with postfusion structures, providing structural snapshots of spike activation. Our results show that receptor binding progressively destabilizes the spike’s fusion machinery and promotes conformational change in a way distinct from SARS-CoV-2, suggesting that different coronaviruses may employ distinct strategies to activate membrane fusion. We also identify a conserved conformational switch that might be important for the final step of membrane fusion, revealing a promising target for pan-coronavirus inhibitor design. Together, these findings improve our understanding of coronavirus entry and provide a structural foundation for developing antiviral agents that target the spike fusion process.

## Introduction

Middle East respiratory syndrome coronavirus (MERS-CoV) is a highly pathogenic zoonotic β-coronavirus that causes severe and often fatal respiratory disease. Since its emergence in 2012 in Saudi Arabia and Jordan, the virus has continued to pose a global health concern, with a case-fatality rate of ~35% [1,2]. Alongside SARS-CoV and SARS-CoV-2, MERS-CoV is one of three highly pathogenic β-coronaviruses to infect humans. Although MERS-CoV is endemic in dromedary camels across the Arabian Peninsula, repeated travel-associated infections and recent detections in humans in Africa suggest a broader ecological and geographic footprint than previously recognized [3–5]. The continued risk of zoonotic spillover, combined with the absence of approved antiviral therapies or vaccines, underscores the need to elucidate the molecular mechanisms underlying MERS-CoV entry and transmission.

β-coronavirus entry is a multistep process driven by receptor-dependent activation of the trimeric spike (S) glycoprotein, a class I viral fusion protein composed of the S1 and S2 subunits. The S1 subunit mediates receptor recognition through its receptor-binding domain (RBD), whereas the S2 subunit contains the membrane fusion machinery [6,7]. For SARS-CoV and SARS-CoV-2, extensive structural and biochemical studies have defined how receptor engagement, proteolytic priming, and S2 refolding coordinate to drive membrane fusion. Structures of the SARS-CoV-2 spike in the prefusion, fusion intermediate, and postfusion states have shown that entry begins when ACE2 engages an RBD in the “up” conformation, which promotes sequential opening of the remaining RBDs. Once all three RBDs adopt the “up” conformation, the S2 subunit undergoes extensive conformational rearrangements to form an extended fusion intermediate in which the fusion peptide is projected toward the host membrane and the S2′ cleavage site becomes exposed. Proteolytic cleavage at the S2’ site is required for membrane fusion, because it enables further conformational rearrangements of the S2 subunit, leading to formation of the HR1–HR2 six-helix bundle that brings the viral and cellular membranes together to complete membrane fusion and genome delivery into the cytoplasm [8–13].

By contrast, the entry mechanism of MERS-CoV remains far less understood. Whereas SARS-CoV and SARS-CoV-2 both use ACE2 as their cellular receptor, MERS-CoV engages dipeptidyl peptidase 4 (DPP4, also known as CD26) [14–17]. High-resolution cryo-EM structures have revealed prefusion-stabilized (GenBank accession number JX869059, residues 18–1,294, with an Arg751Ser mutation to abolish the protease cleavage site) MERS-CoV spike ectodomain trimers in two prefusion states- one-RBD-up and two-RBD-up conformations [18], providing a structural basis for receptor recognition [19–21]. However, how DPP4 binding propagates through the trimer to initiate the transition from the prefusion to the postfusion state remains unclear.

Here, we used cryo‑EM to examine the MERS-CoV spike structures following incubation with DPP4 at defined time points. We identified a series of receptor-bound conformational states spanning prefusion, receptor-bound, and postfusion conformations. These structures show that progressive RBD opening is accompanied by weakening of the S1–S2 interface, followed by S1 dissociation and transition to the postfusion S2 conformation. Together, our findings provide structural insight into receptor-induced activation of the MERS-CoV spike that is distinct from SARS‑CoV‑2 spike under our experimental conditions. suggesting that different coronaviruses may employ distinct pathways of receptor-induced spike activation. These findings broaden our understanding of the mechanistic diversity of coronavirus entry and provide a structural framework for the development of antiviral strategies targeting spike-mediated membrane fusion.

## Results

### Structural transition of MERS Spike upon DPP4 activation

MERS‑CoV spike ectodomain and soluble DPP4 were expressed in HEK293F cells, purified and used for electron microscopy (EM) analysis (S1 Fig). Negative-stain EM showed that, in the absence of DPP4, the spike remained predominantly in the prefusion trimeric state after 24 h of incubation. By 72 h, approximately 92% of the particles remained as prefusion trimers, whereas only 8% exhibited a postfusion-like morphology (S2 Fig). In contrast, incubation of the spike with DPP4 at a 1:1.1 molar ratio resulted in substantially promoted conformational transitions. To examine spike conformations at different incubation times, cryo-EM data were collected after 1 h, 24 h, 72 h, and 120 h of incubation.

At 1 h, three prefusion conformations were identified, corresponding to the 3-down, 1-up, and 2-up states, classified according to the number of RBDs adopting the up conformation (S3 Fig). At 24 h, all four prefusion states (3-down, 1-up, 2-up and 3-up) were observed, together with a very small population (~2%) of postfusion-like particles (S4-S5 Figs). By 72 h, the proportion of prefusion conformations had decreased, whereas postfusion particles had accumulated to a level sufficient for high-resolution reconstruction. In addition, we identified dissociated S1 rings and a putative S2 intermediate (S6-S7 Figs). By 120 h, no intact prefusion trimers were detected, and the sample consisted predominantly of postfusion spikes (S8 Fig). Based on these results, we selected 24 h and 72 h datasets for detailed structural analysis, because they represent distinct conformational populations before and after the emergence of abundant postfusion particles (Fig 1).

**Fig 1:**
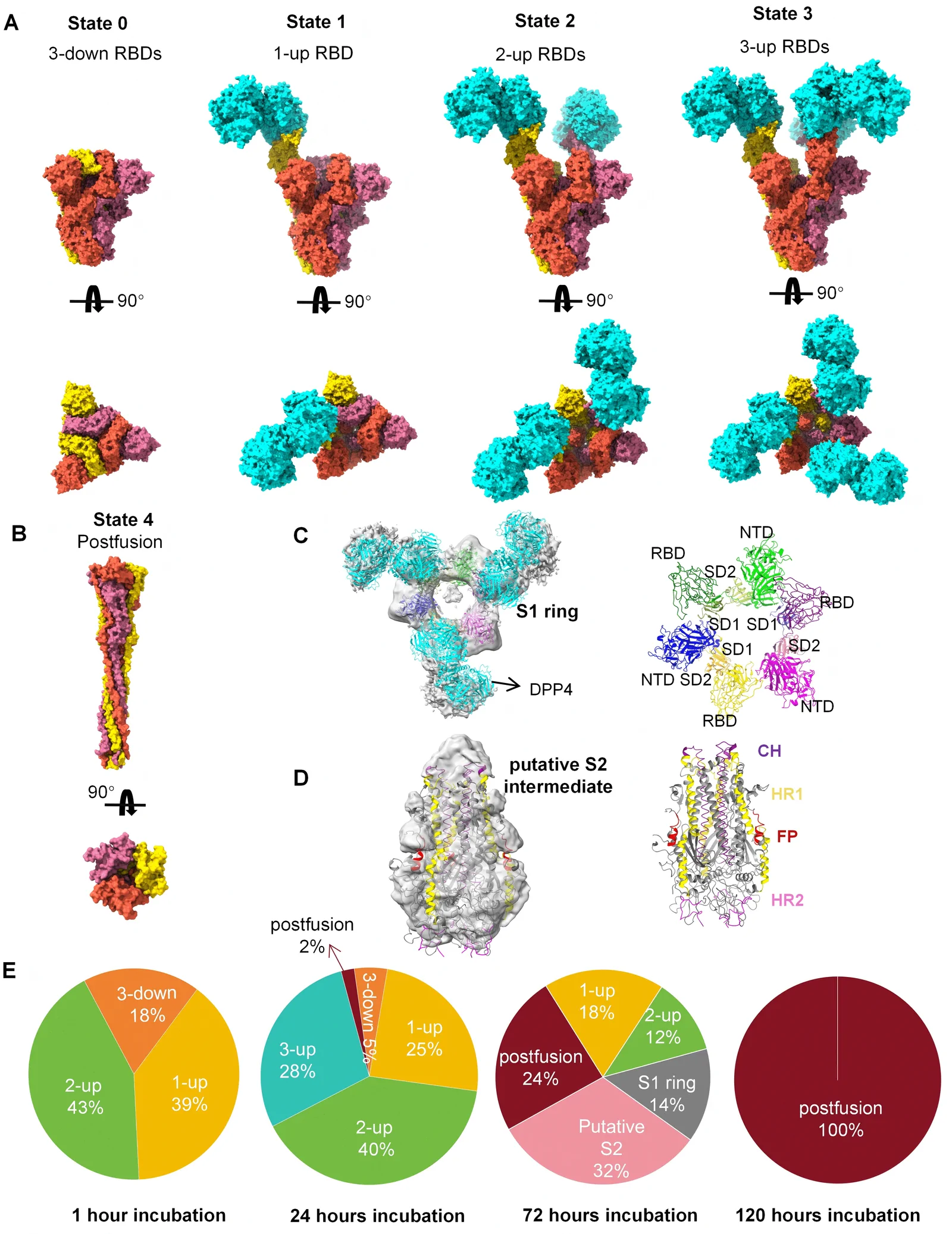
Overall structure of MERS-CoV spike and DPP4 complex. (**A**) Cryo-EM structures of the MERS-CoV S trimer in complex with DPP4 after 24 h of incubation, shown in surface representation. DPP4 is colored cyan. (**B**) Cryo-EM structure of the postfusion S2 conformation observed after 72 h of incubation. (**C-D**) Following 72-hour incubation with DPP4, additional species were observed: dissociated S1 subunits (**C**) and S2 subunits adopting a putative intermediate conformation (**D**). Although these species were not resolved to high resolution, they were clearly discernible in 2D class averages. (**E**) Quantification of prefusion, postfusion, S1 ring, and putative S2 intermediate particles at 1 h, 24 h, 72 h, and 120 h of incubation. Only intact spike trimers were included in the quantitative analysis; RBD–DPP4 regions were excluded since they might be a mixture of different particles.

In addition to intact spike trimers, particles corresponding to the RBD–DPP4 region were observed in all cryo-EM datasets, and an RBD–DPP4 reconstruction was obtained at each incubation time point. Inspection of the corresponding 2D class averages suggests that the contributing particles are heterogeneous. These particles may represent dissociated RBD–DPP4 complexes, flexible receptor-bound spike trimers, or a small fraction of fragmented particles generated during sample preparation; their origin could not be unambiguously determined. Therefore, these particles were excluded from the quantitative analysis, which included only intact spike trimers.

Structural analysis of the MERS-CoV spike-DPP4 complex after 24 h of incubation revealed four distinct conformational states of S trimers (Fig 1A). The majority of spike trimers remained in prefusion conformations, with different combinations of RBDs adopting “up” or “down” positions. A small fraction (~5%) of trimers remained in the unliganded 3-down state. Upon receptor engagement, three classes of spike-DPP4 complexes were identified, containing 1-up (~25%), 2-up (~40%), or 3-up (~28%) RBDs (Fig 1E). In all three classes, each up RBD was bound by a DPP4 dimer.

In contrast, analysis of the MERS-CoV S-DPP4 complex after 72 h of incubation revealed six distinct conformational states (Fig 1E), with a markedly different distribution from that observed at 24 h. Among intact S trimers, the proportion of the 1-up state decreased modestly from 25% to 18%, whereas the 2-up state declined more substantially from 40% to 12%. The 3-up state was no longer detected. In addition, a dissociated MERS-CoV S1 trimer was reconstructed to 5.85 Å resolution (Figs 1C and S6), revealing a ring-like assembly composed of the NTD, RBD, SD1, and SD2 domains, with all three RBDs adopting the “up” conformation [18]. These observations suggest that the fully open (3-up) conformation is intrinsically unstable and prone to S1 dissociation, which may account for the absence of the 3-up state at 72 h. Furthermore, 2D classification revealed a class of S2 particles undergoing conformational rearrangements but not yet fully transitioned to the postfusion state; however, their structural heterogeneity precluded high-resolution reconstruction (Figs 1D and S6). Correspondingly, the proportion of postfusion spikes increased to ~24% of all intact trimers (Fig 1A and 1E).

Taken together, these observations reveal a progressive shift in the conformational landscape of the MERS-CoV spike following DPP4 binding. Receptor engagement is accompanied by the sequential appearance of distinct receptor-bound prefusion conformations, dissociated S1 rings, putative S2 intermediates, and an increasing proportion of postfusion spikes over time. These observations are consistent with DPP4 promoting progressive conformational transitions of the MERS-CoV spike under the experimental conditions used.

### Structures of MERS-CoV S-DPP4 complex

Previous structural studies of the MERS-CoV S-DPP4 complex have been largely confined to the isolated RBD. Cryo-EM analyses of the spike trimer have captured prefusion states with one, two, or three RBDs in the “up” conformation, but all of these were observed in an engineered prefusion-stabilized construct (S-2P) in the absence of DPP4 binding [22]. In contrast, the structure of the wild-type (WT) MERS-CoV spike ectodomain in complex with DPP4 has not previously been reported. One possible explanation is that receptor binding promotes conformational changes that reduce the stability of the prefusion spike. In this study, prolonged incubation with DPP4 enabled us to capture WT spike conformations with one, two, or three RBDs in the “up” position. These receptor-bound conformations exhibited distinct distributions at different incubation time points: at 24 h, the relative abundances followed the order 2-up RBDs > 3-up RBDs > 1-up RBD. Reconstructions of these three major classes reached resolutions of 3.35 Å, 3.44 Å, and 3.29 Å, respectively (S4, S5 Figs).

To investigate the structural basis for the reduced stability of the fully open (3-up) conformation, we compared the S1-S2 interface and interaction networks in the fully closed (3down-RBDs) versus fully open (3-up RBDs) states (taking one protomer as an example). In the 3-down conformation, S1 and S2 bury a substantially larger surface area (6518.3 Å², PISA) (Fig 2A) and appear to form an extensive network of potential hydrogen bonds involving S2 and SD2 residues, as well as between the RBD-NTD connecting loop and S2-CH residue R1057 with the down-RBD (Fig 2C). By contrast, the 3-up state exhibits a smaller buried interface (5293.8 Å²) (Fig 2B), with fewer apparent contacts around the S2 apex (residues 1056–1062), and fewer hydrogen bonds involving the RBD and RBD-NTD loop (Fig 2D). These structural changes are consistent with RBD elevation exposing the receptor-binding surface while weakening S1–S2 interactions, potentially priming the spike for S1 dissociation and subsequent membrane fusion [23].

**Fig 2:**
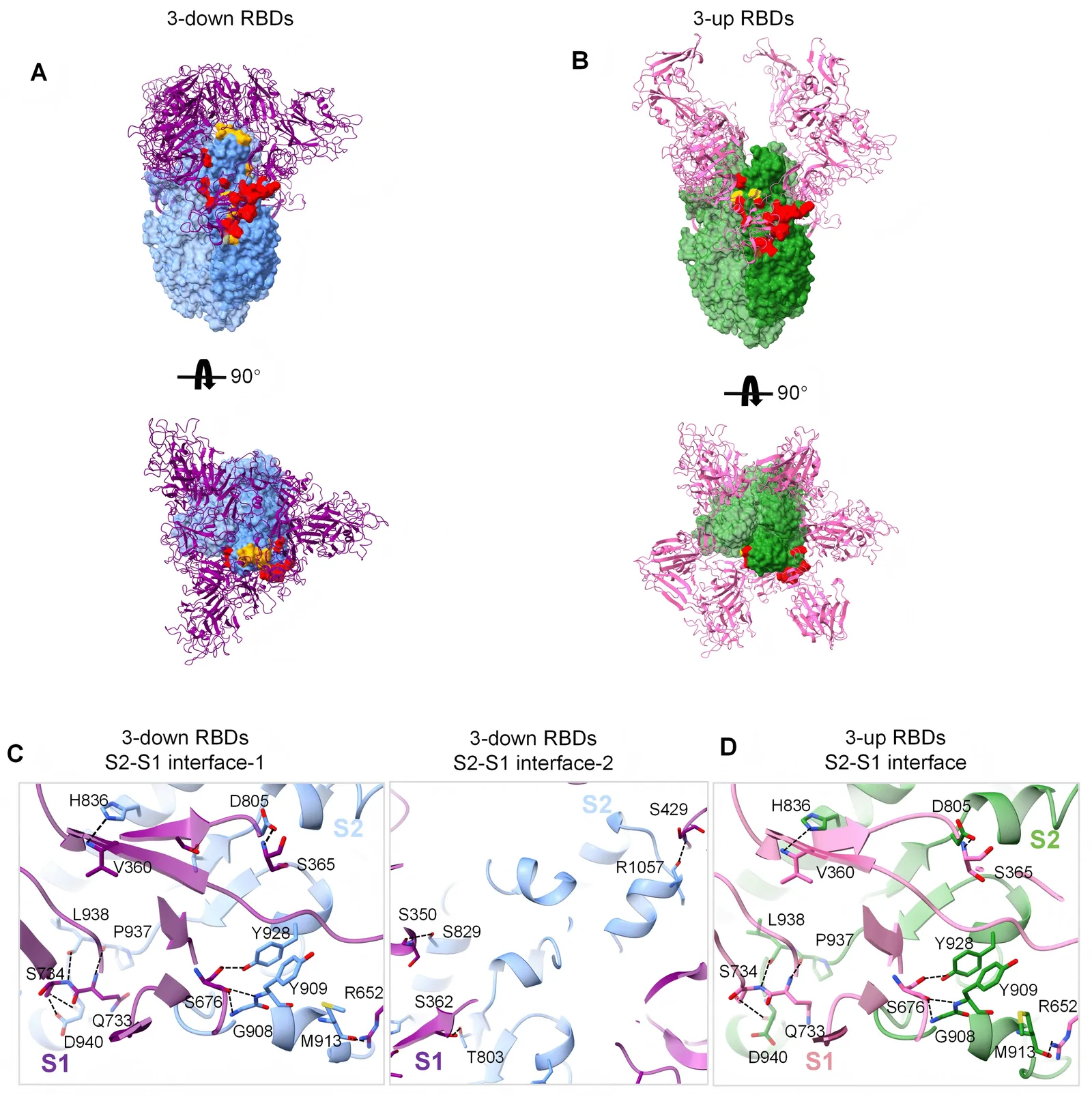
Comparative analysis of S1-S2 interactions between 3-down RBDs and 3-up RBDs states in MERS-CoV. (**A, B**) Comparison of the S1–S2 interfacial surfaces in the all-down RBD conformation (**A**) and the all-up RBD conformation (**B**). Surfaces shared by both conformations are colored red, while surfaces unique to each conformation are colored gold. (**C, D**) Detailed intermolecular interactions between S2 and S1. Hydrogen bonds are depicted as dashed lines. Only interactions between one S2 chain and its neighboring S1 subunit are shown.

Notably, the isolated RBD–DPP4 reconstruction shows density consistent with a DPP4 dimer simultaneously engaging two RBDs. This observation raises the possibility that DPP4 dimers could crosslink neighboring spike trimers by engaging one receptor-accessible RBD from each trimer. If such receptor-mediated clustering occurs in vivo, it could potentially reduce the accessibility of antibody epitopes on the spike, thereby contributing to immune evasion [24].

### Progressive Loss of RBD Stabilization in the 2-Up and 3-Up States

To investigate how RBD elevation may influence trimer stability, we superimposed one “down” RBD from the 3-down, 1-up, and 2-up RBDs conformations and analyzed its interactions with surrounding domains (Fig 3A and 3B). This comparative analysis provides a structural basis for interpreting why the population of the 1-up RBD state remains relatively constant over time, whereas the 2-up RBDs population declines substantially and the 3-up RBDs conformation eventually disappears.

**Fig 3:**
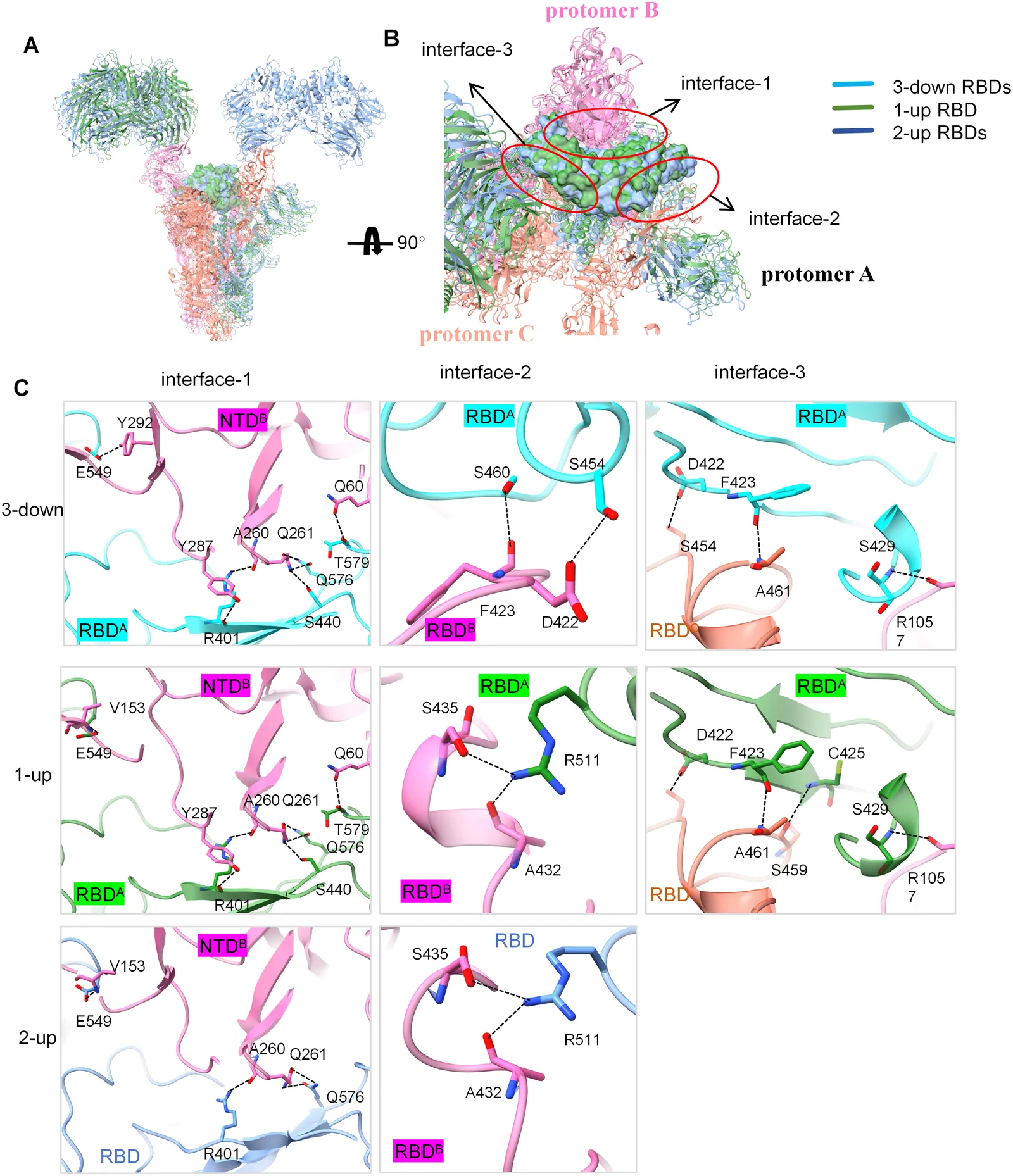
Comparison of the interactions between the down-RBD and the surrounding domains in the three conformations of “3-down, 1-up and 2-up RBDs”. (**A, B**) Alignment of the three conformational states based on the down-RBD, shown in both side and top views. The interactions between the down-RBD and the surrounding domains are classified into three distinct interfaces. The RBD in each of the three conformations is colored Cyan, Green, and Cornflower Blue, respectively. (**C**) Detailed interactions between the down-RBD and adjacent domains across the different conformational states. Hydrogen bonds are represented by dashed lines.

In the 3down-RBD conformation, RBD^A^ appears to be stabilized by a number of hydrogen bonds distributed across three interfaces. At interface 1, putative hydrogen-bonding interactions are observed between RBD^A^ residues R401, S440, E549, Q576, T579 and adjacent NTD^B^ residues Y287, A260, Y292, Q261, Q60. At interface 2, RBD^A^ residues S460 and S454 form two additional hydrogen bonds with RBD^B^ residues F423 and D422. At interface 3, the linear epitope residues D422, F423, and S429 of RBD^A^ form three hydrogen bonds with S454 and A461 of RBD^C^ and with R1057 from protomer B S2 (Figs 3C and S9A). Together, these interactions are consistent with stabilization of RBD^A^ in the “down” conformation.

When RBD^B^ transitions to the “up” state and binds DPP4, the interaction network of the “down” RBD^A^ is modified but remains largely preserved. The contacts at interfaces 1 and 3 are largely preserved, and an additional contact may form between C425 of RBD^A^ and S459 of RBD^C^ at interface 3. At interface 2, although some contacts are lost, new potential interactions may be formed between RBD^A^ residue R511 and S435/A432 on the “up” RBD^B^ (Figs 3C and S9B). Overall, RBD^A^ may participate in a similar or slightly greater number of stabilizing contacts in the 1-up state compared to the 3-down RBDs state, which is consistent with the relatively constant abundance of the 1-up population after 72 h of DPP4 incubation.

In contrast, in the “2-up RBDs” conformation, interactions between the remaining “down” RBD^A^ and the adjacent NTD^B^ are substantially weakened, with only a few potential contacts retained (involving RBD^A^ residues R401, Q576, E549 and NTD^B^ residues A260, Q261, V153). Although new hydrogen bonds form between R511 on RBD^A^’s apical loop and S435/A432 on the “up” RBD^B^, all hydrogen bonds with RBD^C^ are lost (Figs 3C and S9C). This reduction in putative stabilizing interactions is consistent with decreased stability of the remaining down RBD^A^, which may facilitate its transition to the up conformation, thereby permitting engagement of a third DPP4 molecule. The resulting 3-up RBDs state may therefore be less stable, which may contribute to S1 trimer dissociation. Following S1 dissociation, the S2 subunit would be relieved of constraints imposed by S1 and can undergo extensive conformational rearrangements required for transition to the postfusion state.

### Distinct S2-Core Rearrangements in MERS‑CoV and SARS‑CoV‑2

We next compare the S2 region of four prefusion states of MERS‑CoV S and find that the MERS S2 core undergoes progressive conformational drift as more RBDs adopt the “up” conformation. To quantify these changes, the four prefusion states were superimposed using the S2 core CH helix (residues V1060-C1106) as the reference. A central reference axis for the 3-down state was defined in ChimeraX. The distance from the apical residues E1062, conserved across SARS-CoV, SARS-CoV-2 and MERS-CoV S, to this axis was measured for each conformation. Binding of a single DPP4 molecule was accompanied by an outward displacement of the S2 fusion domain. Following engagement of two DPP4 molecules, the HR1-CH region was displaced by 4.2 Å relative to the 3-down conformation, as indicated by the movement of CH residue E1062. For comparison, the same analysis was applied to SARS-CoV-2, using its conserved CH core (residues V987-V1033) and measuring distances from residue E988 to the central axis. In contrast, the SARS-CoV-2 spike exhibited minimal movement of the S2 core even after binding three ACE2 molecules (Figs 4A and S9D).

**Fig 4:**
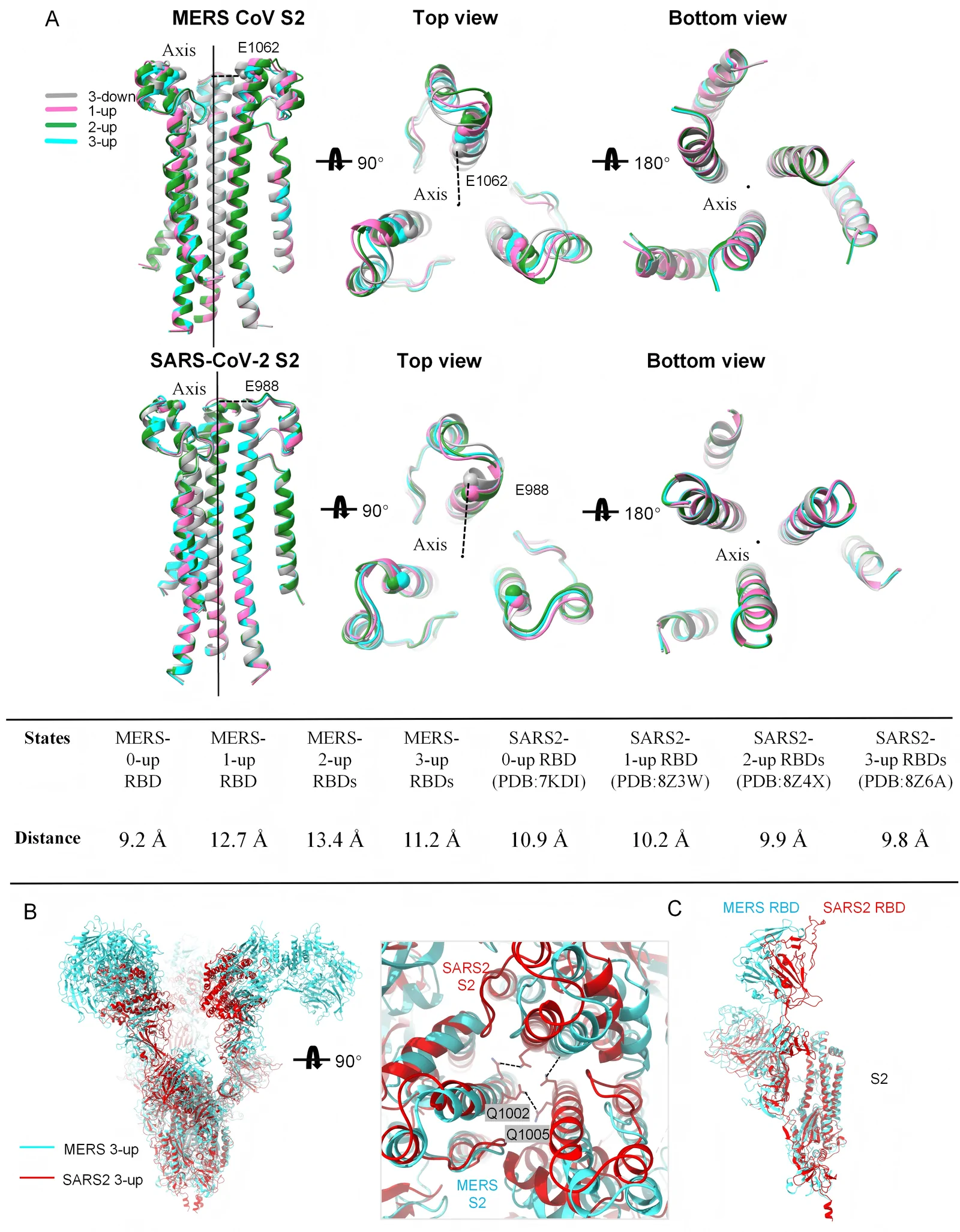
Comparison of the S2 core between MERS CoV and SARS-CoV-2. (**A**) Positional shifts of HR1-CH in the “1-up, 2-up, and 3-up RBD” conformations (following binding of MERS-CoV S to DPP4 and SARS-CoV-2 S to ACE2) were measured relative to its position in the 3-down RBD conformation. The displacement of residue E1062 (E988 in SARS-CoV-2) from the central symmetry axis was quantified across different conformational states. Side, top, and bottom views are shown. The “3-down,” “1-up,” “2-up,” and “3-up” RBD conformations are colored gray, pink, green, and cyan, respectively. (**B**) Comparison of the S2 subunit conformational states between MERS-CoV and SARS-CoV-2 in their 3-up RBD configurations. (**C**) Structural comparison of the MERS-CoV and SARS-CoV-2 RBDs following alignment of the S2 CH regions.

These observations suggest that MERS-CoV and SARS-CoV-2 may employ different strategies for fusion activation. Our structural observations are consistent with a receptor-induced activation model for MERS-CoV, in which the DPP4 binding is accompanied by progressive outward displacement of the S2 core. This displacement may weaken interactions between S1 and S2, promoting S1 dissociation. By comparison, previous studies have suggested that efficient activation of the SARS-CoV-2 spike requires up to three ACE2 receptors [8], thereby preventing premature activation by one or two receptors and ensuring that fusion occurs only under optimal conditions.

Comparison of the conserved CH regions further highlighted structural differences between the two spikes. Superposition of the MERS S trimer bound to three DPP4 molecules and the SARS-CoV-2 S trimer bound to three ACE2 molecules revealed that the MERS S2 core is more loosely packed and contains fewer putative inter-chain hydrogen-bonding interactions within the CH core.

By contrast, the SARS-CoV-2 CH helices form a compact structure stabilized by three pairs of inter-chain hydrogen bonds involving Q1002 and Q1005 (Fig 4B). This more extensive hydrogen-bonding network may contribute to greater structural integrity of the SARS-CoV-2 spike. This structural interpretation is further supported by independent biophysical measurements: differential scanning fluorimetry (DSF) showed that the SARS-CoV-2 D614G spike remains intact after two months at 37 °C [25], whereas the MERS-CoV prefusion spike trimer exhibits a melting temperature of only 58 °C and is noted to be conformationally unstable [26]. Single-molecule FRET also revealed that the MERS-CoV S2 domain is intrinsically dynamic [27], consistent with our observation of S2-core drift upon DPP4 binding. Notably, while spike stability may provide structural basis, the viral transmission efficiency is determined by multiple determinants, including receptor binding affinity and immune evasion. Furthermore, the MERS RBD undergoes a larger upward rotation compared to SARS-CoV-2 (Fig 4C). This more pronounced conformational motion may facilitate S1 dissociation which is consistent with the accumulation of isolated RBD–DPP4 particles observed in our datasets. Together with the observed conformational drift of the S2 core, these findings support a model in which receptor binding progressively destabilizes the MERS-CoV spike under the experimental conditions used.

### Overall structure of MERS-CoV S2 fusion machinery in postfusion state

We determined the cryo-EM structure of the MERS-CoV postfusion S2 subunit at 3.23 Å resolution (S6-S7 Figs). The refined model spans residues S775-Y1288 but lacks the fusion peptide (FP, residues 941–982), its upstream fusion peptide proximal region (FPPR, residues 903–940), and part of the adjacent linker (L, residues 844–900) (Fig 5A). The MERS-CoV postfusion S2 trimer adopts a rod-like architecture approximately 212 Å in length and 34–67 Å in width, closely resembling the postfusion S2 structures of SARS-CoV [28] (PDB: 6M3W), SARS-CoV-2 [29] (PDB: 8FDW) and MHV (PDB: 6B3O) [30] (Fig 5B and 5D).

**Fig 5:**
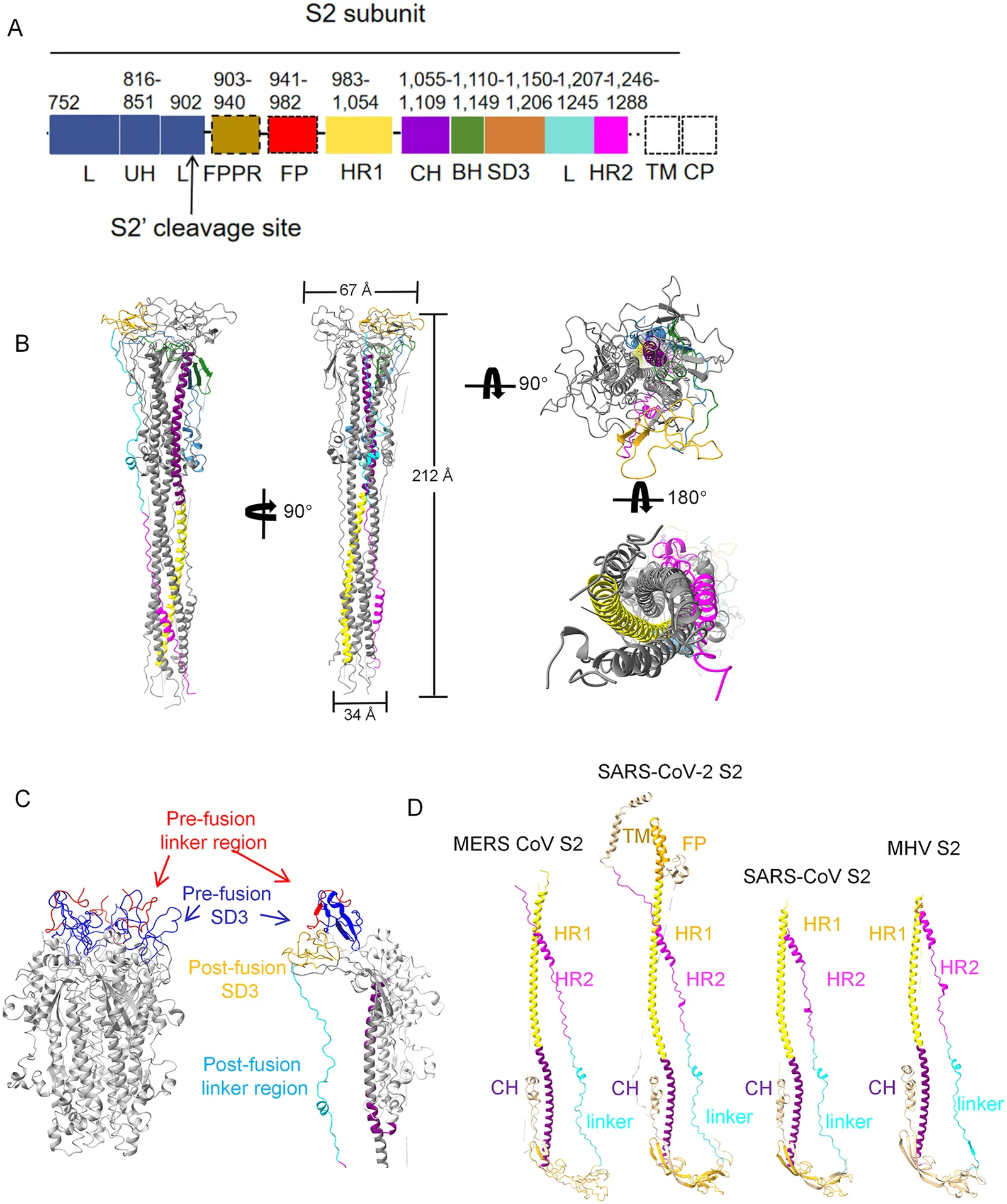
Overall structure of MERS-CoV S2 fusion machinery in postfusion state. (**A**) Schematic diagram of the MERS-CoV S2 subunit ectodomain. The dashed region represents the undetermined region in our structure. L linker region, UH the upstream helices, FP fusion peptide, FPPR fusion peptide proximal region, HR heptad repeat, CH central helix, BH β-hairpin, SD3 subdomain. (**B**) Cartoon representation of the MERS-CoV S2 trimer in postfusion state colored as shown in A. Overall structural changes of the linker region upstream of the HR2 motif during membrane fusion. (**C**) The linker regions in pre-fusion and postfusion states are shown as cartoons colored in red and cyan, respectively. The SD3 domains in prefusion and postfusion states are shown as cartoons colored in blue and gold, respectively. (**D**) Comparison of MERS-CoV (colored as A), SARS-CoV-2 (PDB: 8FDW), SARS-CoV (PDB:6M3W) and MHV (PDB:6B3O) postfusion S2 subunits in single chain mode.

The postfusion S2 is characterized by a long central helical bundle flanked by shorter α-helices and β-strands at the membrane-distal end. The HR1 and central helix (CH) motifs fold into a continuous α-helix, with three HR1-CH long helices coiling around each other to form the stalk region of the trimer. The HR2 motif and its upstream linker (L) pack antiparallel into grooves formed between two adjacent HR1-CH helices, while the β-hairpin (BH) motif and subdomain 3 (SD3) are situated around the C-terminal end of the central HR1-CH helices, partially capping the top of the central helical core (Fig 5B).

Notably, the linker upstream of HR2, which is only partially ordered in the pre-fusion states of both SARS-CoV [18] and MERS-CoV spikes, becomes largely ordered in the postfusion state. These structural observations suggest that the HR2 upstream linker may play an important role during membrane fusion. In the pre-fusion state, a β-strand from this linker forms a β-sheet with the SD3 domain, stabilizing it in a folded conformation adjacent to SD3. By contrast, in the postfusion state, the linker undergoes a dramatic structural rearrangement: it dissociates from SD3, reverses orientation, and refolds into an extended, loop-rich structure that binds along the grooves of the stem helices from neighboring protomers (Fig 5C). This structural transformation is likely required for proper assembly of the central HR1-HR2 six-helix bundle, which is thought to generate the force required to draw the viral and host membranes into close proximity during the final stage of membrane fusion. The HR2 upstream linker may serve as a structural relay, potentially coupling receptor-induced conformational changes to HR1–HR2 six-helix bundle assembly during membrane fusion.

These observations suggest that stabilizing the prefusion conformation of the HR2 upstream linker, or preventing its rearrangement, may interfere with membrane fusion, highlighting this region as a potential target for the development of broad-spectrum antivirals and neutralizing antibodies. A recent study identified a hydrophobic “lock” centered on residue F1148 within the SARS-CoV-2 HR2 upstream linker that is required for efficient six-helix bundle assembly and membrane fusion, as mutations at this site impair fusion activity [31]. Structural comparison of the postfusion S2 subunits revealed that MERS-CoV F1231 corresponds to SARS-CoV-2 F1148 and resides in a structurally equivalent hydrophobic cavity (S10 Fig). This observation raises the possibility that a similar hydrophobic lock contributes to the refolding of the MERS-CoV S2 subunit. These observations are consistent with a conserved functional role of the HR2-linker region across β-coronaviruses.

Although the FPPR and FP are not resolved in our MERS-CoV structure, their positions can be inferred from the SARS-CoV-2 postfusion structure, in which the CH and HR1 regions form a continuous helix that is predicted to position the fusion peptide for insertion into the host membrane (Fig 5D). In the postfusion state, the HR1 and HR2 regions assemble into the characteristic six-helix bundle, which is thought to bring the viral and host membranes into close proximity during the final stage of membrane fusion.

Comparative analysis of the postfusion S2 structures of MERS-CoV, SARS-CoV, and SARS-CoV-2 revealed a highly conserved domain reorganization (S11 Fig). Although the FPPR and FP are not resolved in our MERS-CoV structure, their positions can be inferred from the SARS-CoV-2 postfusion structure, in which the CH and HR1 regions form an exceptionally long, continuous helix that is predicted to position the fusion peptide for insertion into the host cell membrane (Fig 5D). In the postfusion state, the HR1 and HR2 regions assemble into the characteristic six-helix bundle, which brings the viral and host membranes together to complete membrane fusion and enable viral infection.

## Discussion

Our study provides structural insight into the receptor-induced activation of the MERS-CoV spike by capturing a series of receptor-bound conformational states spanning prefusion, receptor-bound, and postfusion states. Although our analysis is based primarily on two incubation time points (24 h and 72 h), with additional observations at 1 h and 120 h, the structures reveal distinct conformational populations that outline a plausible pathway of receptor-induced spike activation. Because these datasets represent discrete structural snapshots rather than continuous kinetic measurements, our conclusions are limited to comparisons of the conformational states enriched under the experimental conditions used.

Previous studies have shown that the WT MERS-CoV spike in the absence of receptor can adopt multiple prefusion conformations: 3-down, 1-up, and 2-up RBDs [18]. Our data further show that prolonged incubation with DPP4 results in the appearance of the fully open 3-up state, followed by S1 dissociation. Receptor binding is accompanied by conformational changes in the S2 core and a progressive reduction in stabilizing interactions surrounding the remaining down RBD, which may facilitate its transition to the up conformation. When all three RBDs adopt the “up” conformation, the interactions between S1 and S2 are weakened, facilitating the dissociation of S1 trimer ring. The S2 subunit subsequently refolds into the characteristic six-helix bundle, drawing the viral and host membranes together to complete membrane fusion (Fig 6).

**Fig 6:**
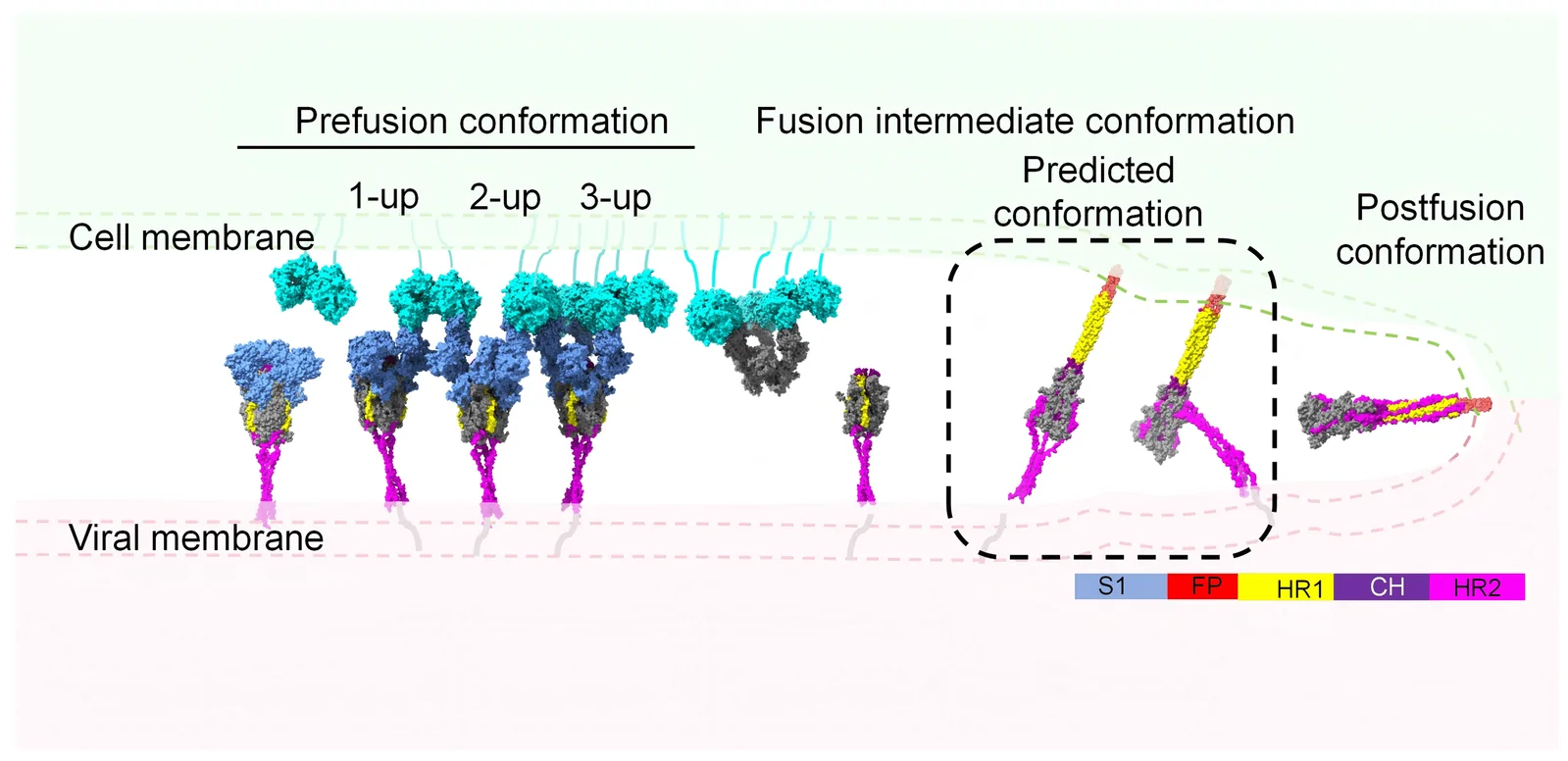
MERS-CoV S protein undergoes conformational changes during membrane fusion. These models were constructed by superimposing the “1-up, 2-up, 3-up RBDs” S trimer with the RBD-DPP4 complex structure (through the RBD domain). The S trimer of the MERS-CoV can interact with the dimeric form of the DPP4 receptor during the binding process. When the three RBDs are simultaneously activated, the interaction between S1 and S2 also significantly weakens. S2’s position becomes displaced and loose, which is equivalent to “pushing” S1 from the inside. The S1 ring falls off, and S2 begins to undergo intense rearrangement. The connecting region, HR1 region and central helix will form an extremely long helix, inserting the fusion peptide into the host cell membrane. Finally, the HR1 and HR2 regions will form a helical structure and assemble into a six-helix bundle, pulling the viral membrane and the host membrane together to complete the infection.

Our structural observations suggest that MERS-CoV and SARS-CoV-2 employ distinct receptor-induced activation pathways. Xing et al. recently reported an early fusion intermediate (E-FIC) of the SARS-CoV-2 spike within 10 min after ACE2 binding under their experimental conditions [8], whereas the MERS-CoV spike remained predominantly in the prefusion state after 24 h in our in vitro system, with abundant postfusion particles appearing only after prolonged incubation (72 h). We did not capture an E-FIC analogous to that reported for SARS-CoV-2 under our experimental conditions (25 °C, soluble proteins, and no membrane environment), this does not exclude the existence of a similar intermediate in MERS-CoV, which may require different experimental conditions to stabilize. Although these studies used different experimental conditions, the available structural data qualitatively suggest that receptor-induced conformational progression is slower in MERS-CoV than in SARS-CoV-2. One possible explanation is that the SARS-CoV-2 S1 ring remains associated during early fusion, whereas receptor binding progressively weakens the S1-S2 interface in MERS-CoV, resulting in premature S1 dissociation before complete S2 refolding. Consistent with these structural observations, SARS-CoV-2 also exhibits faster replication kinetics than MERS-CoV in cultured cells and reaches peak viral loads earlier in infected patients [32]. However, viral replication kinetics are influenced by multiple factors beyond spike activation, including receptor distribution, protease availability, tissue tropism, and host immune responses. Whether these structural differences contribute to the distinct biological properties of the two viruses remains to be established.

Despite these differences, the postfusion S2 architecture is highly conserved among MERS-CoV, SARS-CoV and SARS-CoV-2. In particular, the HR2 upstream linker undergoes a pronounced conformational rearrangement from a β-sheet to an extended loop. Together with its structural conservation across β-coronaviruses, this rearrangement suggests that the HR2 linker may represent a potential target for the development of broad-spectrum fusion inhibitors and neutralizing antibodies.

Our isolated RBD-DPP4 reconstruction also revealed a DPP4 dimer simultaneously engaging two RBDs. Although our data do not provide direct evidence that a single DPP4 dimer bridges two intact spike trimers, they raise the possibility that receptor-mediated crosslinking could occur on the cell surface. If present in vivo, such interactions might influence spike organization or antibody accessibility and therefore warrant further investigation.

The conformational progression observed in our cryo-EM experiments occurred over a much longer timescale than viral entry in infected cells. This difference most likely reflects the deliberately simplified in vitro conditions used here, in which purified spike ectodomain and soluble DPP4 were incubated at 25°C in the absence of membranes, proteases, endosomal acidification, and other host factors known to facilitate coronavirus entry. Although we cannot directly establish that the conformational states observed in vitro correspond to those formed during infection, the sequence of structures captured here is broadly consistent with current models of β-coronavirus spike activation and membrane fusion.

In summary, this study captures representative conformational states of the MERS-CoV spike at defined incubation times, spanning receptor binding, progressive RBD opening, S1 dissociation, and postfusion refolding. Although an early fusion intermediate analogous to that described for SARS-CoV-2 was not observed under our experimental conditions, its existence in MERS-CoV cannot be excluded and may require different experimental conditions to stabilize. Together, these findings define a receptor-induced activation pathway that differs in several respects from that of SARS-CoV-2, expanding our understanding of coronavirus entry and providing a structural framework for future mechanistic studies and antiviral development.

## Methods

### Protein expression and purification

The gene encoding the ectodomain of the MERS-CoV spike (UniProt: K9N5Q8, residues 1–1,291) was synthesized and subcloned into a mammalian expression vector pCMV vector containing an N-terminal gp67 signal peptide, a C-terminal T4 fibrin trimer domain and 6X His tag. The plasmid was transfected into HEK293F cells by polyethylenimine, supernatant was harvested after 72h, and the S trimer or DPP4 was purified with the Ni-Sepharose resin from Cytiva Reagents [33]. The target protein was further purified by Superose 6 Increase 10/300 column (GE Healthcare) in 20 mM Tris, 200 mM NaCl, pH 8.0 buffer (S1 Fig). SDS-PAGE was used to detect protein size and purity [34].

### Cryo-EM data collection and processing

MERS-CoV spike was diluted to 0.7 mg/mL and mixed with 1.5 mg/mL DPP4 at a molar ratio of 1:1.1 (S trimer/DPP4), and incubated for 24 h at 25°C. The 3 μL composite was added to a new light-emitting porous amorphous Ni-Ti alloy film supported by 400 mesh gold mesh. The sample was immersed in liquid ethane using Vitrobot IV (FEI, Thermo Fisher Scientific) and frozen for 2.0 s, a blot force of -3, and a wait time of 10.0 s. Cryo-EM data for MERS S-DPP4 were automatically collected using EPU software on a Titan Krios G4 microscope at 300 kV. The device was equipped with Falcon 4i and Seletris X-ray imaging filters (Thermo Fisher) with slit width set to 20 eV. EER movie stacks were acquired in super-resolution mode with nominal magnification of 130000x for MERS-CoV S-DPP4, corresponding to a physical pixel size of 0.932 Å and a dose of 1080 frames [7]. The defocus range was from -1.0 to -3.0 μm, and the total dose was approximately 50e^−^ /A^2^. All data processing was performed using Relion (v3.1) and cryoSPARC (v4.4.1). Movie stacks were motion‑corrected in Relion using MotionCor2 [35] with dose weighting, followed by CTF estimation using Gctf [36]. All micrographs were then imported into cryoSPARC for further processing. Particle picking was carried out using both blob picker and template picker. Picked particles were subjected to at least two rounds of 2D classification, then merged and duplicates removed. Ab‑initio models were generated in cryoSPARC using selected 2D class averages. For classes with few particles, Topaz training was performed to improve particle picking. After merging and duplicate removal, heterogeneous refinement was applied. Each class then underwent two independent rounds of 3D classification. Clean particles were selected and subjected to non‑uniform refinement in cryoSPARC to obtain 3D reconstructions. Local refinement was performed for regions with lower resolution, using masks generated in Relion and then imported into cryoSPARC. The final resolutions obtained were: 3‑down (3.24 Å), 1‑up (3.30 Å), 2‑up (3.00 Å), 3‑up (3.44 Å, C3 symmetry), RBD‑DPP4 (3.27 Å), and postfusion (3.23 Å). The S1 ring and the putative S2 intermediate were resolved at lower resolutions (5.85 Å and 6.60 Å, respectively).

The reported resolution is based on the gold standard Fourier shell correlation (FSC) of 0.143. The data-processing algorithm is summarized in S3-S8 Figs.

To evaluate potential bias introduced by the Topaz model, we used the high‑resolution map of each resolved conformational state as a template to train independent Topaz models. Particles picked by each model were merged, duplicates were removed, and the combined particle set was subjected to heterogeneous refinement to automatically assign particles to the respective classes and recalculate their proportions. The workflows for calculating the particle distribution of conformational states at different time points are shown in the corresponding supplementary figures: 1 h (S3 Fig), 24 h (S4 Fig), 72 h (S6 Fig), and 120 h (S8 Fig).

### Model building and refinement

EMready [37] was used to generate sharpened maps for model building, SWISS-MODEL [38] was used to generate initial prediction models, UCSF Chimera [39] was used to fit models into maps and manually adjust them in COOT [40], followed by multiple rounds of real space refinement in PHENIX [41]. Models were validated using PHENIX. Figures were created by UCSF Chimera and UCSF ChimeraX [42]. Statistics of data collection and model refinement are shown in S1 Table.

## Supporting information

S1 Fig Gel filtration curve of the non-lysed MERS-CoV-WT S-terminal domain trimer and the DPP4 homodimer. The MERS-CoV-WT S trimer remained stable during gel filtration, which was confirmed by SDS-PAGE.
(TIF)

S2 Fig Workflow of negative-stain EM image processing for the receptor-free MERS-CoV S protein at 24 h (A) and 72 h (B). (TIF)

S3 Fig Cryo-EM data processing workflow for the MERS-CoV S-DPP4 complex at the 1 h. (TIF)

S4 Fig Cryo-EM data processing, density maps, and particle distribution workflow for the MERS-CoV S-DPP4 complex at 24 h. This figure integrates the data processing workflow, representative density maps of major conformations, and the particle distribution calculation for the 24 h dataset. Density maps were rendered in UCSF ChimeraX and colored according to local resolution estimated by cryoSPARC (FSC = 0.143).
(TIF)

S5 Fig Gold-standard FSC curves (top) and particle orientation distribution of MERS-CoV S complexed with DPP4 after 24-hour incubation. (TIF)

S6 Fig Cryo-EM data processing, density maps, and particle distribution workflow for the MERS-CoV S-DPP4 complex at 72 h. This figure integrates the data processing workflow, representative density maps of major conformations, and the particle distribution calculation for the 72 h dataset. Density maps were rendered in UCSF ChimeraX and colored according to local resolution estimated by cryoSPARC (FSC = 0.143).
(TIF)

S7 Fig Gold-standard FSC curves (top) and particle orientation distribution of MERS-CoV S complexed with DPP4 after 72-hour incubation. (TIF)

S8 Fig Cryo-EM data processing workflow for the MERS-CoV S-DPP4 complex at the 120 h time point. (TIF)

S9 Fig Local resolution density maps supporting the structural interpretations in Figs 3 and 4. (A) Representative cryo-EM density for key RBD interface residues from the 3‑down conformation (Fig 3). (B) Representative density for key residues involved in the 1‑up state at the RBD interface (Fig 3). (C) Representative density for key residues in the 2‑up state at the RBD interface (Fig 3). (D) Density for the S2 core CH helix region and residue E1062 from Fig 4.
(TIF)

S10 Fig Structural comparison of the hydrophobic “lock” in the HR2 upstream linker between SARS-CoV-2 and MERS-CoV postfusion structures. (A) Superimposed postfusion S2 subunits of SARS-CoV-2 (yellow, PDB: 8FDW) and MERS-CoV (cyan, PDB: 22EC). (B) Detailed view of SARS-CoV-2 F1148 (red) embedded in the hydrophobic cavity formed by surrounding residues. (C) Detailed view of MERS-CoV F1231 (blue) occupying a structurally equivalent hydrophobic cavity. The similar hydrophobic environment suggests that a conserved hydrophobic lock may drive six‑helix bundle formation and membrane fusion across β‑coronaviruses.
(TIF)

S11 Fig S2 sequence alignment of MERS-CoV, SARS-CoV, and SARS-CoV-2. (TIF)

S1 Table Cryo-EM data collection and refinement statistics. Note: Structures of 3-down RBDs, 1-up RBD, 2-up RBDs, 2-up RBDs local, 3-up RBDs, and DPP4-RBD were determined from the 24 h dataset; the postfusion structure was determined from the 72 h dataset.
(PDF)

S1 Raw Gel Original, uncropped SDS-PAGE gel images corresponding to S1 Fig. This file contains the original, uncropped, and unadjusted SDS-PAGE gel images for the SDS-PAGE shown in S1 Fig.
(PDF)
