# Supplementary material for: Receptor-induced activation of MERS-CoV spike revealed by time-course cryo-EM
Source: PLoS Pathog. 2026 Oct 8;22(10):e1014545. doi: 10.1371/journal.ppat.1014545 (PMC13648974; doi:10.1371/journal.ppat.1014545)
Supplement: Supplementary file 1 [file ppat.1014545.s012.pdf]

S1 Table . Cryo-EM data collection and refinement statistics.

	3-down RBDs	1-up RBD	2-up RBDs	2-up RBDs local	3-up RBDs	DPP4-RBD	Post-fusion
**Data collection and processing**							
Magnification	130,000						
Voltage (kV)	300						
Total dose (e⁻/Å²)	50						
Defocus range (μm)	-1.0 to -3.0						
Pixel size (Å)	0.932						
Symmetry imposed	C3	C1	C1	C1	C1	C1	C3
Final particles (no.)	6625	28966	188119	188119	44232	21290	93300
Map Resolution (Å)	3.24	3.29	3.35	3.4	3.44	3.27	3.23
**Model Refinement**							
**R.m.s. deviations**							
Bond lengths (Å)	0.002	0.001	0.002	0.002	0.001	0.002	0.002
Bond angles (°)	0.526	0.386	0.427	0.606	0.369	0.546	0.472
**Validation**							
MolProbity score	2.32	1.96	1.95	2.40	1.65	2.11	2.30
Clash score	6.15	6.08	5.55	7.13	5.44	5.19	5.26
Rotamer outlier (%)	5.29	2.65	2.85	4.96	1.65	6.29	5.23
**Ramachandran plot**							
Favored (%)	93.47	95.6	95.66	92.14	96.86	96.52	92.32
Allowed (%)	6.36	4.40	4.30	7.61	3.12	3.16	7.68
Disallowed (%)	0	0	0	0	0	0	0
**EMDB**	EMD-68252	EMD-68188	EMD-68189	EMD-68190	EMD-68191	EMD-68192	EMD-68193
**PDB**	22FY	22DX	22DY	22DZ	22EA	22EB	22EC

Note: Structures of 3-down RBDs, 1-up RBD, 2-up RBDs, 2-up RBDs local, 3-up RBDs, and DPP4-RBD were determined from the 24 h dataset; the postfusion structure was determined from the 72 h dataset.
