# Supplementary material for: Receptor-induced activation of MERS-CoV spike revealed by time-course cryo-EM
Source: PLoS Pathog. 2026 Oct 8;22(10):e1014545. doi: 10.1371/journal.ppat.1014545 (PMC13648974; doi:10.1371/journal.ppat.1014545)

This image corresponds to Figure 1A

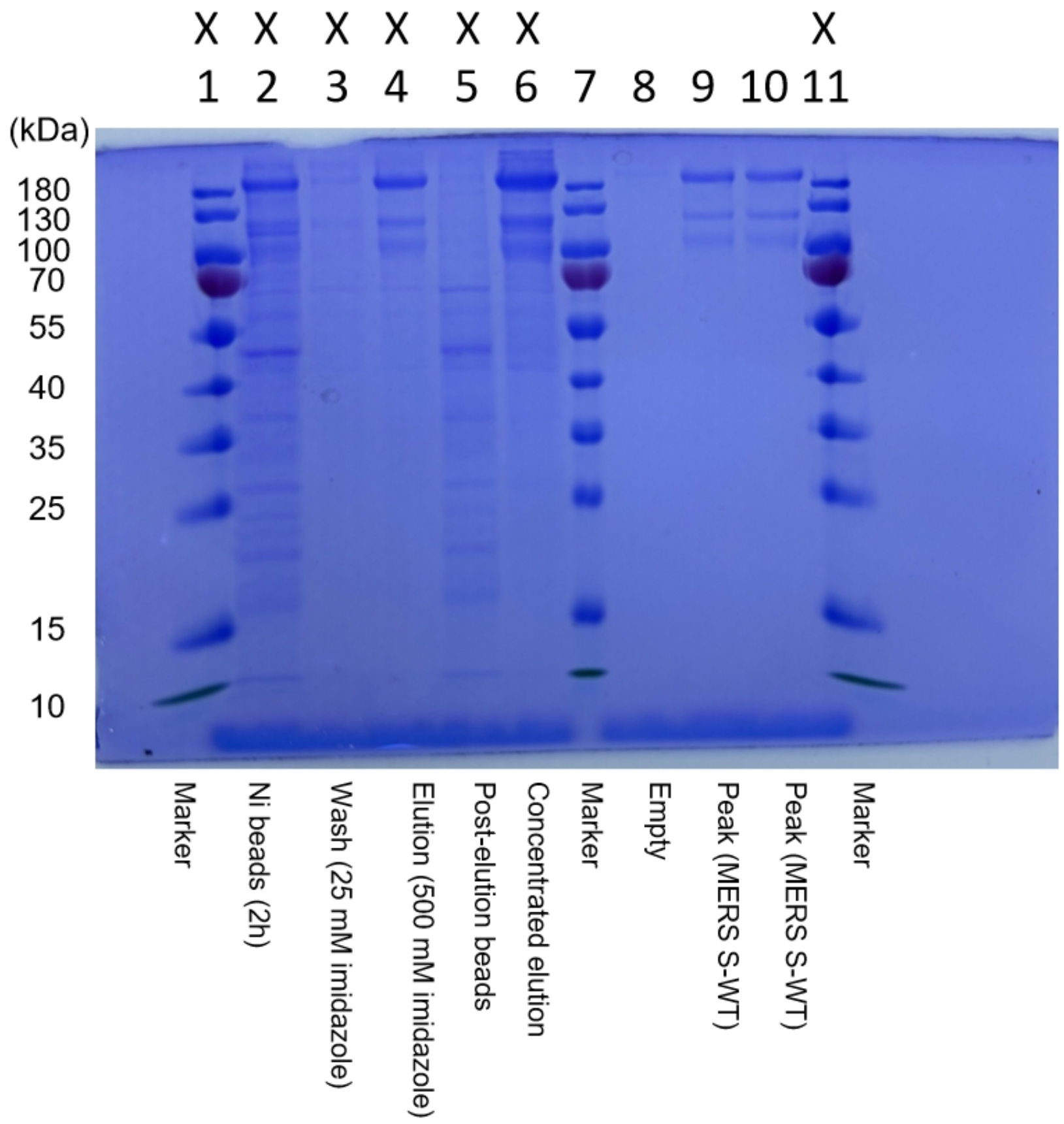

This image corresponds to Figure 1B

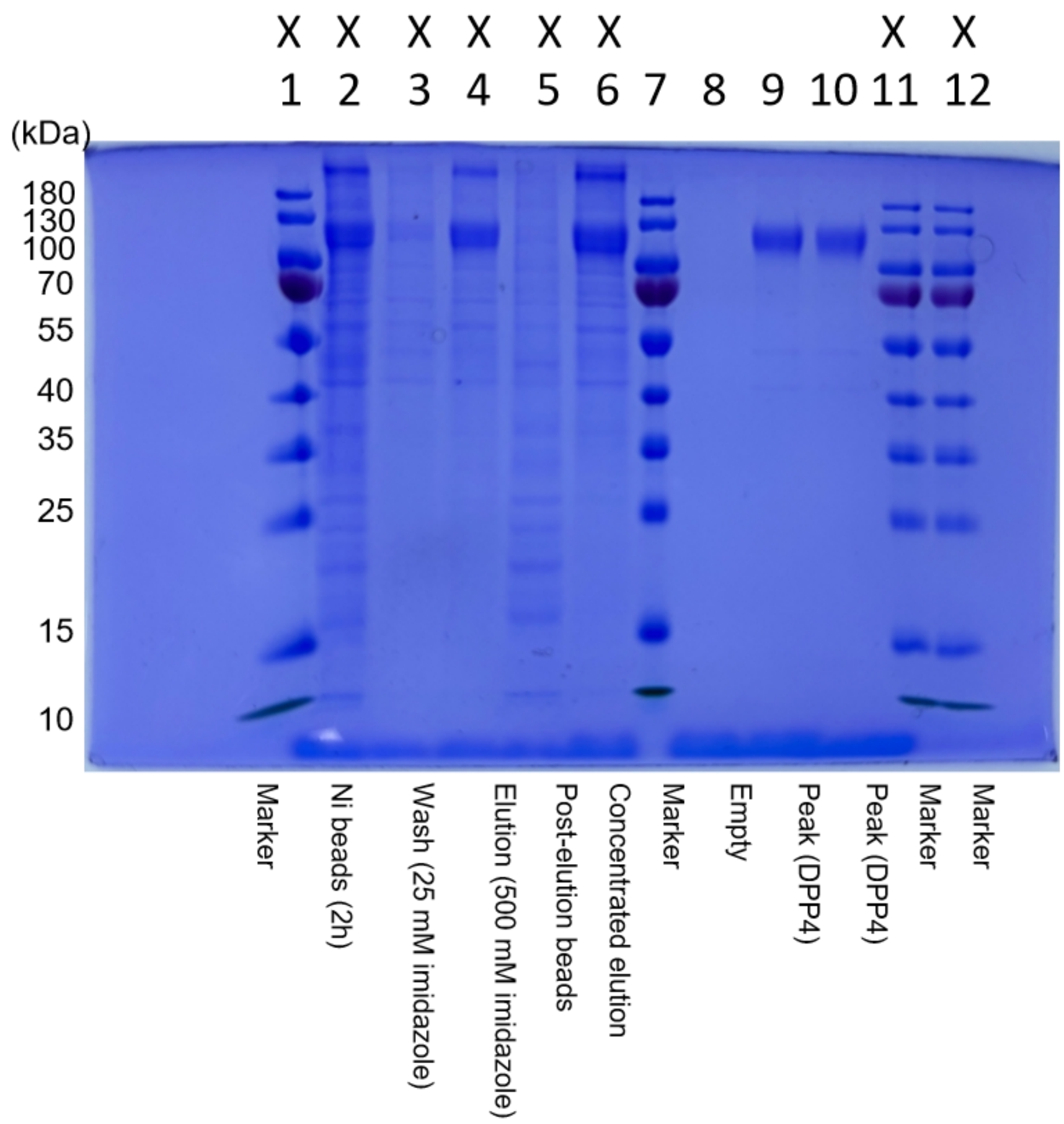

Supplement: Supplementary file 2 [file ppat.1014545.s013.pdf]
