# Supplementary material for: Receptor-induced activation of MERS-CoV spike revealed by time-course cryo-EM
Source: PLoS Pathog. 2026 Oct 8;22(10):e1014545. doi: 10.1371/journal.ppat.1014545 (PMC13648974; doi:10.1371/journal.ppat.1014545)
Supplement: Supplementary file 3 [file ppat.1014545.s015.pdf]

Additional Editor Comments:

As you will see from the reviewers reports, several important concerns have been raised. In particular, all three reviewers agree that the manuscript overstates its conclusions and employs mechanistic language that is not fully supported by the data. The characterization of the study as “time-resolved” (including the **title**) is considered misleading, as it relies on only two widely separated time points (24 and 72 hours) and does not capture true kinetics or continuous dynamics. There is also a consistent concern regarding the lack of functional or experimental validation to support key claims, including those related to stability, activation mechanisms, and biological relevance.

In addition, the robustness and reproducibility of the structural analysis are questioned in several instances. The authors are asked to provide clearer methodological details, including validation of classification approaches and evidence of reproducibility. Furthermore, the manuscript does not sufficiently engage with the existing literature, as relevant studies are sometimes not cited and previously reported findings are presented as novel. Finally, the resolution of the maps does not support the level of detail used to describe specific interactions, particularly hydrogen bonds, and these interpretations should be presented more cautiously.

Response: We thank the Editor and reviewers for their constructive comments. We have comprehensively revised the manuscript to address all concerns. The title and abstract no longer use "time-resolved," and we have systematically moderated mechanistic language throughout (e.g., "demonstrates" → "*suggests*" "critical switch" → "*may act as*"). We added new data including receptor-free controls (Fig S2), 1h and 120h time points (Fig S3 and Fig S8), local-resolution density maps for key residues (Fig S9), and structural comparisons with SARS-CoV-2 and MHV. We have also provided detailed methodological descriptions, cross-software validation of classification approaches, and added eight relevant citations to better engage with the literature. All hydrogen-bond interpretations have been presented more cautiously, and we explicitly acknowledge the limitations of our two-time-point design in the Discussion. We believe the revised manuscript is now more rigorous, precise, and suitable for publication.

Response to Reviewers

Reviewers' Comments:

Part I - Summary

Please use this section to discuss strengths/weaknesses of study, novelty/significance, general execution and scholarship.

Reviewer #1: In this article, Wang and colleagues study the mechanism of DPP4-mediated activation of the MERS spike using cryo-EM at two different time points, 24 and 72 hours. They report structures of the prefusion (3 RBDs down), intermediate (3, 2, and 1 RBD-up), and postfusion states, allowing them to reconstruct a trajectory of spike activation and to observe how receptor binding weakens the interactions between S1 and S2. The results presented in this article are consistent with what has been previously described for other coronaviruses, help improve our understanding of MERS entry mechanisms, and may have implications for the development of antiviral therapies. I only have a few comments that I hope will help improve the manuscript.

Reviewer #2: In this manuscript, Wang et al describe cryoEM structures of the MERS-CoV spike incubated for various amounts of times with the DPP4 receptor. Although the premise of the work is interesting, the manuscript provides limited new insights and is not suitable as is for publication.

Reviewer #3: This study by Wang et al. is an interesting structural manuscript that substantially advances our understanding of MERS-CoV spike activation. The authors capture receptor-bound 1-up, 2-up, and 3-up prefusion states and determine a postfusion S2 structure. Overall, I found the study novel, informative, and appropriate for the readership of PLOS Pathogens. My main reservation is not the quality or interest of the dataset, but rather that some of the mechanistic language is stronger than necessary. In particular, terms such as "time-resolved," "direct receptor-triggered fusion mechanism," and some of the more detailed interpretations of residue-level interactions would benefit from softening. That said, these issues do not undermine the core value of the work. I would therefore support publication after revision.

Response: We thank all three reviewers for their thoughtful assessments. We are encouraged that Reviewers #1 and #3 recognize the value of our work, with Reviewer #3 stating that the study "substantially advances our understanding of MERS-CoV spike activation" and is "novel, informative, and appropriate" for *PLOS Pathogens*.

We also appreciate Reviewer #2's acknowledgment that the premise is interesting.

Regarding the shared concerns about overinterpretation and mechanistic language, we have systematically revised the manuscript throughout—moderating claims, removing "time-resolved" from the title/abstract, softening residue-level interaction descriptions, and adding qualifiers such as "*suggests,*" "*may,*" and "*is consistent with.*" We have also strengthened the manuscript with new data (receptor-free controls, 1h and 120h time points, local-resolution density maps) and expanded methodological details to address robustness concerns. We believe these revisions address the reviewers' reservations while preserving the core scientific contributions of our study.

.

Part II – Major Issues: Key Experiments Required for Acceptance
Please use this section to detail the key new experiments or modifications of existing experiments that should be absolutely required to validate study conclusions.

Generally, there should be no more than 3 such required experiments or major modifications for a "Major Revision" recommendation. If more than 3 experiments are necessary to validate the study conclusions, then you are encouraged to recommend "Reject".

Reviewer #1: 1. The authors mention that they use "time-resolved" cryo-EM, but they only show structures of discrete states rather than continuous dynamics. What is the rate (speed) of the reaction? They analyze structures after 24 and 72 hours of incubation with a slight molar excess of receptor, which suggests slow activation kinetics. However, MERS infection experiments (for example, Coleman and Frieman, 2015) show replication products within 12 hours. How can these two observations be reconciled? The authors should include functional data showing the kinetics of receptor-spike interaction, for example using BLI, or data showing that postfusion forms only appear after days of incubation.

Response: We thank the reviewer for raising this important point. We agree that the term "time-resolved" may imply observation of continuous progression of spike activation. Since our study compares structural populations obtained after defined incubation periods rather than capturing continuous kinetic transitions, we have replaced "time-resolved" with "time-course" (or "analysis at different incubation times") throughout the manuscript to more accurately reflect the experimental design. The title now reads: "Receptor-induced activation of MERS-CoV spike revealed by time-course cryo-EM". In the abstract and main text, we now refer to "analysis at different time points (mainly at 24 h and 72 h)".

We acknowledge the reviewer's suggestion regarding functional kinetic measurements. We would like to note that techniques such as BLI primarily quantify receptor–spike binding kinetics (association and dissociation rates), whereas the focus of the present study is the subsequent conformational activation process that occurs after receptor engagement. The structural transitions characterized here involve large-scale rearrangements of the spike protein and are not directly measurable by conventional binding assays.

In addition, the experimental system used in this study consists of purified soluble spike ectodomain and soluble DPP4 under non-physiological conditions (25 °C, pH

8.0, without membranes, proteases, or other cellular cofactors). Under these conditions, the observed structural transitions likely reflect a slow equilibrium redistribution among conformational states rather than the physiological fusion kinetics occurring during viral entry. That is the reason that our time scale is different from that observed in infection experiments (Coleman and Frieman, 2015). We have now explicitly discussed these differences in the revised Discussion section (lines 395-403) as follows: "*The conformational progression observed in our cryo-EM experiments occurred over a much longer timescale than viral entry in infected cells. This difference most likely reflects the deliberately simplified in vitro conditions used here, in which purified spike ectodomain and soluble DPP4 were incubated at 25 °C in the absence of membranes, proteases, endosomal acidification, and other host factors known to facilitate coronavirus entry. Although we cannot directly establish that the conformational states observed in vitro correspond to those formed during infection, the sequence of structures captured here is broadly consistent with current models of β-coronavirus spike activation and membrane fusion.*"

Our objective was to capture and structurally characterize intermediate states along the receptor-induced activation pathway. To address the reviewer's concern regarding the timing of postfusion formation, we re-examined the 24 h dataset and identified a small population (~2%) of postfusion-like particles based on 2D classification, providing evidence that postfusion conversion had already begun by 24 h and became substantially enriched by 72 h (24%). These new data are now presented in Fig. S4 and discussed in the revised manuscript.

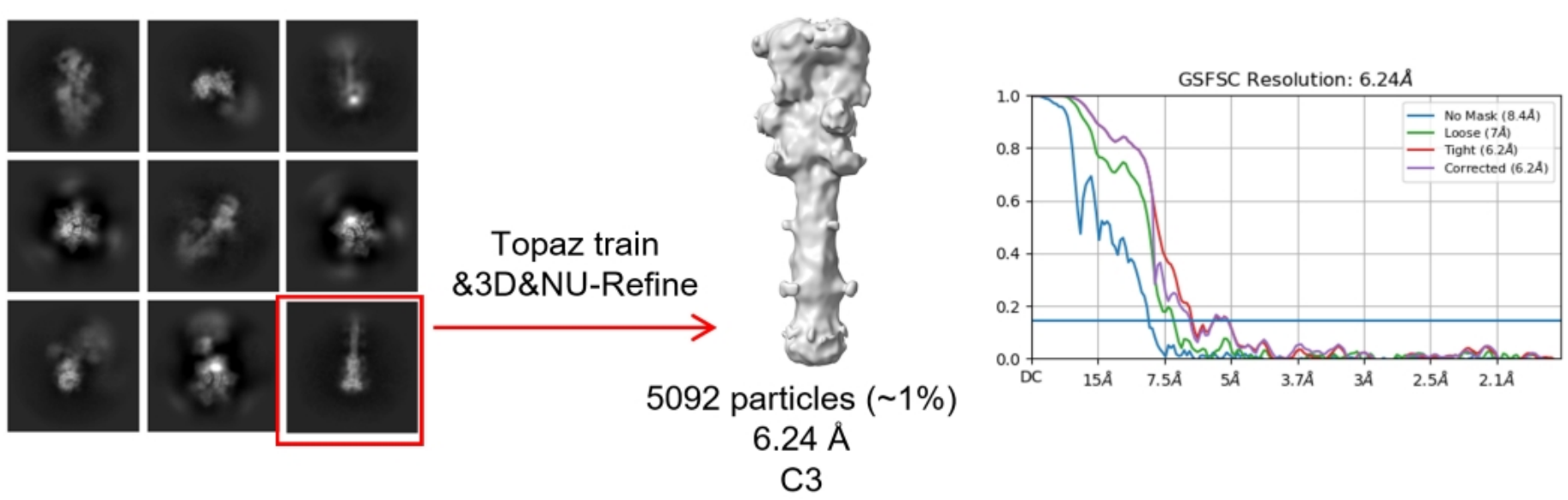

Author Response Figure R1. 2D class classification and 3D reconstruction of the 24 h cryo-EM dataset reveals a small fraction (~2%) of rod-like particles resembling the postfusion S2 conformation.

2. What is the distribution of spike conformations at 24 h and 72 h in the absence of receptor? And what is the distribution in the presence of receptor at 1 h?

Response: We thank the reviewer for raising this question. To address this concern, we performed additional control experiments and analyses, which have now been

incorporated into the revised manuscript.

1. Receptor-free controls (24 h and 72 h)

Purified MERS-CoV S protein (without DPP4) was incubated under the same conditions (25 °C, pH 8.0 buffer) for 24 h and 72 h, and examined by negative-stain EM. At 24 h, almost all particles remained as prefusion trimers. At 72 h, approximately 92% of the particles were still present as prefusion trimers, while only 8% displayed a postfusion-like morphology, corresponding to a prefusion to postfusion ratio of roughly 11:1. In contrast, in the 72 h sample incubated with DPP4, the prefusion-to-postfusion ratio was approximately 1.25:1 (Fig. 1E). These observations indicate that, although a small degree of spontaneous conversion occurs during prolonged incubation, DPP4 binding substantially promotes conformational progression toward the postfusion state. These control data have now been incorporated into the main text (lines 103-107) and are presented in Fig. S2, as follows: "*Negative-stain EM showed that, in the absence of DPP4, the spike remained predominantly in the prefusion trimeric state after 24 h of incubation. By 72 h, approximately 92% of the particles remained as prefusion trimers, whereas only 8% exhibited a postfusion-like morphology (Figure S2).*"

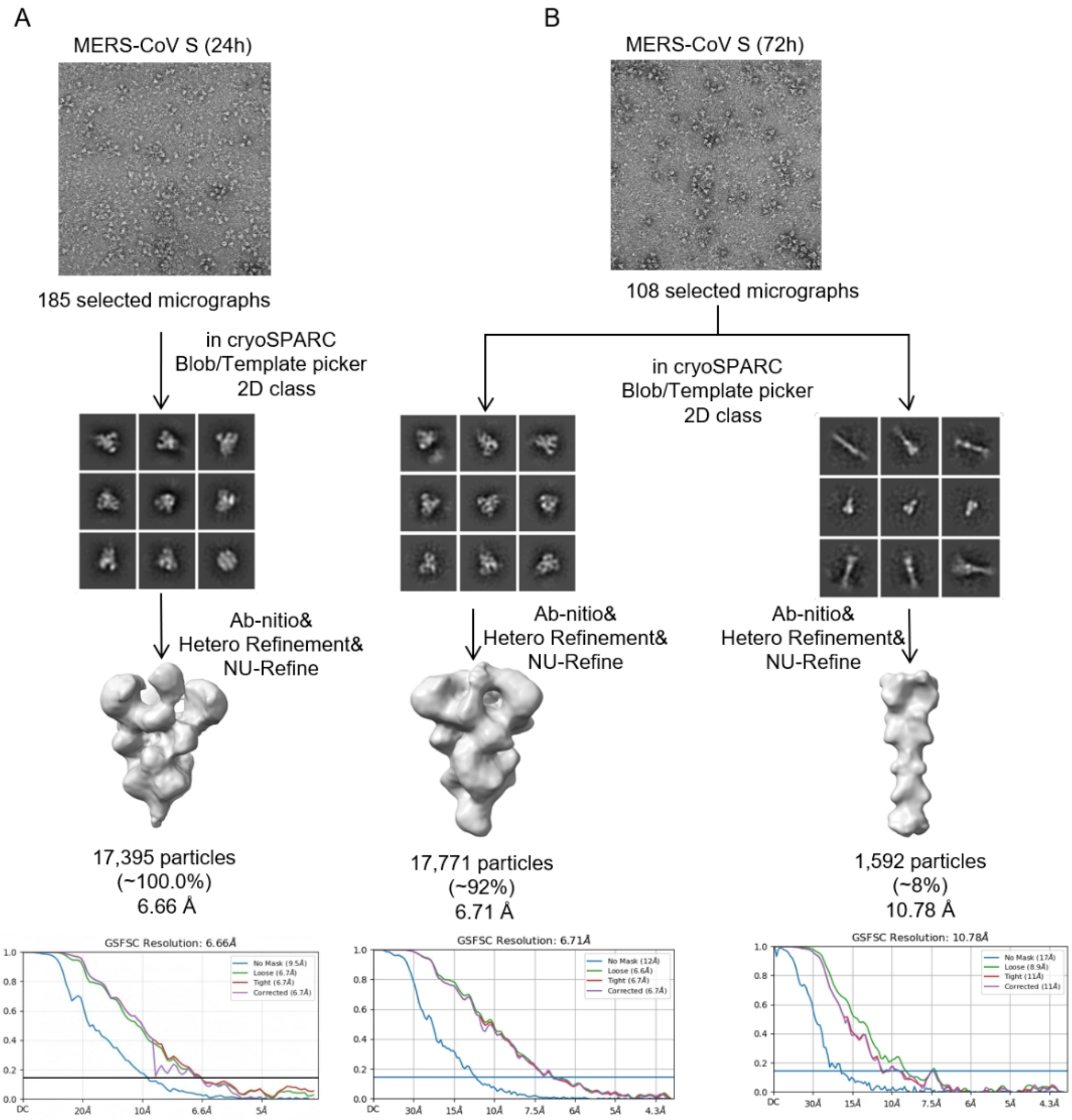

Fig. S2. Workflow of negative-stain EM image processing for the receptor-free MERS-CoV S protein at 24h and 72 h.

2. Conformational distribution at 1 h in the presence of receptor

We also analyzed cryo-EM data collected after 1 h of incubation with DPP4 (now presented in Fig. S3). At this time point, three prefusion trimers are classified: 3-down RBDs, 1-up RBD, 2-up RBD states, whereas no 3-up RBDs state was detected. The 1-up and 2-up conformations were present at approximately equal proportions, while the 3-down RBDs state was less abundant. This distribution contrasts with those differs from those observed at later time points, where the 2-up state predominates at 24 h and abundant postfusion particles appear at 72 h, and is consistent with progressive receptor-induced opening of the RBDs over time.

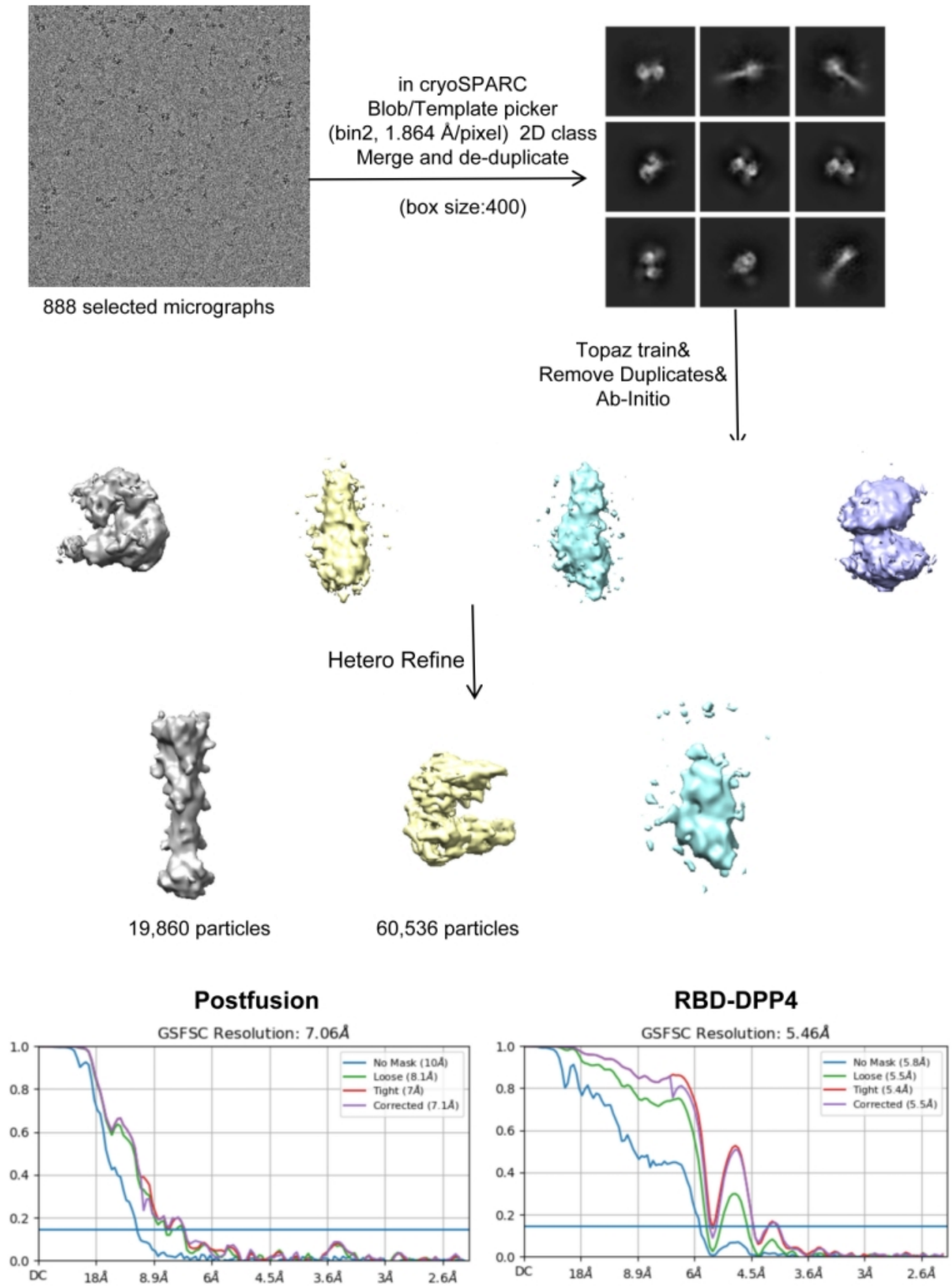

Fig. S3. Cryo-EM data processing workflow for the MERS-CoV S-DPP4 complex at the 1 h time point.

Conclusion: The receptor-free controls experiments indicate that spontaneous conversion to postfusion-like particles occurs only at a low level under our experimental conditions, whereas DPP4 binding substantially promotes conformational progression toward the postfusion state. In addition, the 1 h cryo-EM dataset incule more 3-down confirmation and no 3-up RBDs state. Together, these observations are consistent with a receptor-promoted, stepwise activation pathway of the MERS-CoV spike.

3. One possibility is that the observed differences are due to artifacts related to sample preparation or cryo-EM data processing. How reproducible are these results?

Response: We thank the reviewer for raising this important concern. We acknowledge that independent replicate grid preparations were not performed for the 24 h and 72 h samples, and therefore technical reproducibility represents a limitation of the present study. Nevertheless, several independent observations support the robustness of our structural conclusions.

Fig. S8. Cryo-EM data processing workflow for the MERS-CoV S-DPP4 complex at the 120 h time point.

In addition to the 24 h and 72 h datasets, we analyzed samples incubated under the same conditions for 1 h and 120 h (Fig S3 and Fig S8). All four datasets (1 h, 24 h, 72 h, and 120 h) were prepared, vitrified, and processed using the same experimental and image-processing procedures. Across the datasets collected at these four incubation time points, we observed a consistent progression in the distribution of spike conformations: receptor-bound 1-up and 2-up states were present at 1 h, the 2-up state predominated and the 3-up state appeared at 24 h, postfusion S2 became readily detectable at 72 h, and by 120 h the sample consisted almost entirely of postfusion S2 , with no prefusion trimers detected. In addition, receptor-free control samples remained predominantly in the prefusion trimeric state under identical incubation conditions. Together, these observations argue against spontaneous sample degradation or systematic bias arising from sample preparation or image processing as the primary explanation for the observed conformational changes.

We have now included these control data as new supplementary figures in the revised manuscript (lines 108-122), as follows: "*To examine spike conformations at different incubation times, cryo-EM data were collected after 1 h, 24 h, 72 h, and 120 h of incubation. At 1h, three prefusion conformations were identified, corresponding to the 3-down, 1-up, and 2-up states, classified according to the number of RBDs adopting the up conformation (Figure S3). At 24 h, all four prefusion states (3-down, 1-up, 2-up and 3-up) were observed, together with a very small population (~2%) of postfusion-like particles (Figure S4-S5). By 72 h, the proportion of prefusion conformations had decreased, whereas postfusion particles had accumulated to a level sufficient for high-resolution reconstruction. In addition, we identified dissociated S1 rings and a putative S2 intermediate (Figure S6-S7). By 120 h, no intact prefusion trimers were detected, and the sample consisted predominantly of postfusion spikes (Figure S8). Based on these results, we selected 24 h and 72 h datasets for detailed structural analysis, because they represent distinct conformational populations before and after the emergence of abundant postfusion*

particles (Figure 1)."

4. How robust is the particle distribution analysis shown in Figure 1? The authors perform 3D classification using relion, would they obtain the same distribution using cryosparc? To select particles, they train a topaz model to identify the intermediate states. How dependent are the results on this topaz model? How many 3D classes do they use in relion during classification, and how does the final result depend on the number of classes used? The authors should be more explicit about these details in the Materials and Methods section.

Response: We thank the reviewer for raising this important question. We apologize that the original Materials and Methods section did not sufficiently describe our image-processing workflow, which may have caused confusion. We have now substantially expanded the Methods (Line 426-464) and added the following clarifications.

(1) Image-processing workflow

The cryo-EM datasets were processed using both RELION (v3.1) and cryoSPARC (v4.4.1). Movie stacks were motion-corrected in RELION using MotionCor2 (binning factor 2) with dose weighting, followed by CTF estimation using Gctf. The corrected micrographs were then imported into cryoSPARC for particle picking, 2D classification, ab initio reconstruction, heterogeneous refinement, and non-uniform refinement. Local refinement was performed using masks generated in RELION and imported into cryoSPARC. We have revised the Materials and Methods section to clearly describe this workflow.

(2) Consistency between RELION and cryoSPARC.

To assess whether the particle distributions depended on the processing software, we also performed an independent 3D classification analysis in RELION using the same datasets. Both software packages consistently identified the major particle populations, including prefusion spikes and postfusion spikes However, cryoSPARC heterogeneous refinement provided better separation of the closely related prefusion conformations (3-down, 1-up, 2-up, and 3-up), which was therefore used for the final structural analysis and particle quantification.

(3) Potential bias introduced by Topaz particle picking.

Topaz was used only to improve particle picking for low-abundance conformational states and was not used for particle classification. For classes with few particles, Topaz training was performed to improve particle picking. After merging

and duplicate removal, heterogeneous refinement was applied. Each class then underwent two independent rounds of 3D classification. Clean particles were selected and subjected to non-uniform refinement in cryoSPARC to obtain 3D reconstructions. Local refinement was performed for regions with lower resolution, using masks generated in Relion and then imported into cryoSPARC.

To evaluate whether the particle distributions were dependent on the Topaz models, we trained independent Topaz models using each resolved conformational state as the training template. The particles picked by these independent models were merged, duplicates were removed, and the resulting particle set was subjected to the same heterogeneous refinement and classification procedure. Importantly, assignment of particles to individual conformational states was determined exclusively during cryoSPARC heterogeneous refinement and subsequent 3D classification, rather than by the Topaz models themselves. The percentages of individual classes changed slightly after reprocessing, and we have updated these in the manuscript. We also updated the data processing workflow in Fig S4 and S6.

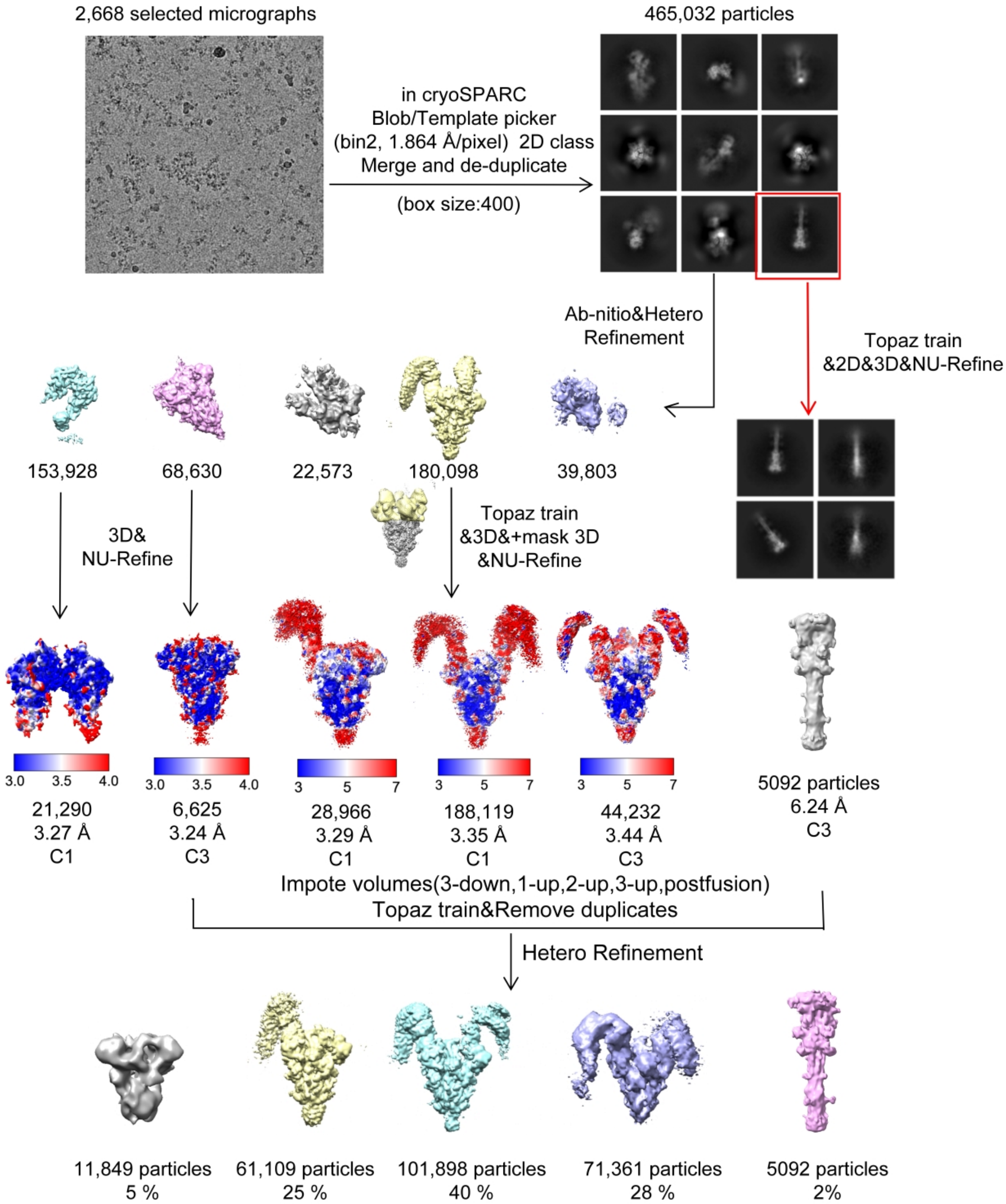

Fig S4 Cryo-EM data processing, density maps, and particle distribution workflow for the MERS-CoV S-DPP4 complex at 24 h.

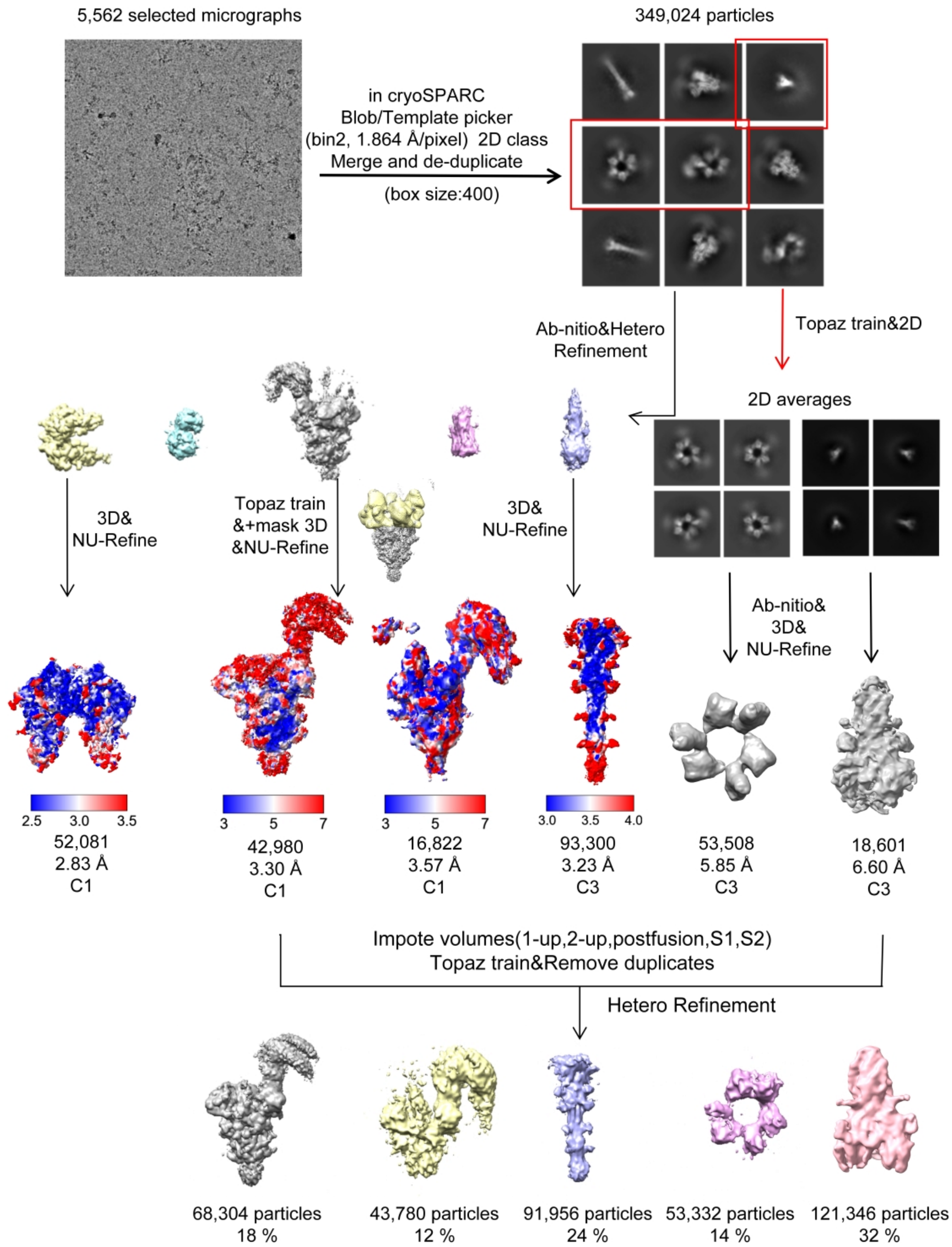

Fig S6 Cryo-EM data processing, density maps, and particle distribution workflow for the MERS-CoV S-DPP4 complex at 72 h.

5. The authors discuss structural differences relative to the SARS-CoV-2 spike. How do the kinetics of activation compare between the two? Are the infection kinetics of the two viruses also different?

Response: We thank the reviewer for raising this insightful question. We agree that comparing the activation and infection kinetics of MERS-CoV and SARS-CoV-2

provides useful context for interpreting our findings. We have therefore expanded the Discussion to address these points.

Activation kinetics:

Xing et al. (Cell, 2025) reported the appearance of an early fusion intermediate (E-FIC) within 10 min after incubation of the SARS-CoV-2 spike with ACE2 at 4 °C (pH 8.0), whereas in our study the MERS-CoV spike, incubated with soluble DPP4 at approximately 25 °C (pH 8.0), remained predominantly in prefusion conformations at 24 h, with abundant postfusion particles appearing only after prolonged incubation (72 h). Although these studies employed different protein constructs, receptors, temperatures, and experimental workflows, the available structural data qualitatively suggest that receptor-induced conformational progression occurs more rapidly in the SARS-CoV-2 spike than in the MERS-CoV spike under the respective in vitro conditions.

A notable structural difference may help explain this contrast. In the SARS-CoV-2 E-FIC, ACE2 undergoes an approximately 6.2 Å downward movement while remaining associated with S1, suggesting that receptor-bound S1 may continue to participate in the fusion process. In contrast, our structures indicate that receptor binding progressively weakens the S1–S2 interface, leading to S1 dissociation before complete S2 refolding. Although the functional consequences of this difference remain to be established experimentally, premature S1 dissociation may reduce the ability of S1 to stabilize or coordinate subsequent conformational changes, thereby contributing to the slower accumulation of postfusion spikes observed in our in vitro system.

Infection kinetics:

Previous studies indicated that the infection kinetics of the two viruses differ. SARS-CoV-2 replication products can be detected within 6–12 h after infection in cultured cells (Hou et al., 2020), whereas MERS-CoV replication is generally detected somewhat later (approximately 12–24 h) (Coleman and Frieman, 2015). In patients, SARS-CoV-2 viral loads typically peak within 3 days after symptom onset, whereas MERS-CoV viral loads generally peak at 7–10 days (Cevik et al., 2021). However, viral replication kinetics are determined by multiple factors beyond spike activation, including receptor distribution, protease availability, and host immune responses, etc. Therefore, although the structural differences described here may contribute to the distinct biological behaviors of the two viruses, they should not be interpreted as the sole determinants of their infection kinetics.

We have added a discussion of these points in the revised manuscript on lines

362-382 as following: "*Xing et al. recently reported an early fusion intermediate (E-FIC) of the SARS-CoV-2 spike within 10 min after ACE2 binding under their experimental conditions*[8]*, whereas the MERS-CoV spike remained predominantly in the prefusion state after 24 h in our in vitro system, with abundant postfusion particles appearing only after prolonged incubation (72 h). Although these studies used different experimental conditions, the available structural data qualitatively suggest that receptor-induced conformational progression is slower in MERS-CoV than in SARS-CoV-2. One possible explanation is that the SARS-CoV-2 S1 ring remains associated during early fusion, whereas receptor binding progressively weakens the S1-S2 interface in MERS-CoV, resulting in premature S1 dissociation before complete S2 refolding. Consistent with these structural observations, SARS-CoV-2 also exhibits faster replication kinetics than MERS-CoV in cultured cells and infected patients. However, viral replication kinetics are influenced by multiple factors beyond spike activation, including receptor distribution, protease availability, tissue tropism, and host immune responses. Whether these structural differences contribute to the distinct biological properties of the two viruses remains to be established.*"

Reviewer #2: 1. The abstract is misleading as it states that the manuscript describes structures obtained though "time-resolved series" including an "intermediate". No such intermediate is described.

Response: We thank the reviewer for pointing out the imprecise wording in the abstract. We agree that the term "time-resolved series" is inaccurate, as our study examines structural populations at discrete incubation times rather than capturing continuous temporal progression. Accordingly, we have revised the title, abstract, and main text to replace "time-resolved" with "time-course" or to explicitly refer to analyses performed at defined incubation times.

Besides, we agree that the term "intermediate "could imply a transient state which is not directly demonstrated by our data. To avoid overinterpretation, we have removed the term "intermediate" from the abstract and replaced it with a more descriptive statement: "*… spanning prefusion, receptor-bound states, and postfusion states.*" Corresponding changes have been made throughout the manuscript to ensure consistent.

2. The authors claim that the SARS-CoV-2 spike is highly stable as compared to the MERS-CoV spike but no experimental measurements are provided to support this claim.

Response: We thank the reviewer for raising this concern. We agree that our original

statement regarding the relative stability of the SARS-CoV-2 and MERS-CoV spikes was stronger than warranted by the data presented in the original manuscript. In the revised manuscript, we have therefore moderated the wording and included more analysis based on structural comparison and the published literature.

First, our structural comparison (Fig.4A) shows that receptor binding is accompanied by a measurable displacement of the MERS-CoV S2 core, whereas the corresponding SARS-CoV-2 S2 core remains largely unchanged in the published receptor-bound structures. Although these structures were obtained in different studies and therefore do not constitute a direct quantitative comparison, they are consistent with the SARS-CoV-2 spike exhibiting a more rigid S2 core than the MERS-CoV spike under the respective experimental conditions.

Second, we have incorporated published biophysical measurements that support this interpretation. Differential scanning fluorimetry studies have shown that the SARS-CoV-2 spike exhibits high thermal stability(Yang et al., 2021), whereas the MERS-CoV prefusion spike has a reported melting temperature of approximately 58 °C and has been described as conformationally unstable. In addition, recent single-molecule FRET studies demonstrated that the MERS-CoV spike spontaneously samples multiple prefusion conformations in S2 region even in the absence of receptor, indicating substantial intrinsic conformational flexibility. Although these measurements were not performed under identical experimental conditions and therefore should not be interpreted as a direct quantitative comparison, they are consistent with the structural differences observed in our study.

We have incorporated the relevant references into the revised manuscript (line 262-270) , as follows: "*This more extensive hydrogen-bonding network may contribute to greater structural integrity of the SARS-CoV-2 spike. This structural interpretation is further supported by independent biophysical measurements: differential scanning fluorimetry (DSF) showed that the SARS-CoV-2 D614G spike remains intact after two months at 37 °C[25], whereas the MERS-CoV prefusion spike trimer exhibits a melting temperature of only 58 °C and is noted to be conformationally unstable[26]. Single-molecule FRET also revealed that the MERS-CoV S2 domain is intrinsically dynamic[27], consistent with our observation of S2-core drift upon DPP4 binding.*"

3. Lines 67-70: the authors seem to suggest that processing of MERS-CoV S2' leads to S1 shedding. What mechanism do the authors envision would lead to this? Why is S1 shedding requiring S2' proteolytic cleavage given that S is already cleaved at S1/S2. The consensus in the field is that S2' cleavage promotes membrane fusion (e.g.

PMID: 27791014, PMID: 25288733).

Response: We thank the reviewer for pointing out this ambiguity. We apologize for the confusing wording in the original manuscript. We did not intend to suggest that S2' cleavage directly causes or is required S1 shedding. We agree with the reviewer that these are distinct events. As demonstrated in previous studies (PMIDs: 27791014, 25288733), proteolytic cleavage at the S2' site promotes the conformational rearrangements of S2 subunit to drive membrane fusion. In contrast, S1 shedding results from destabilization of the S1–S2 interface following receptor engagement and is not, by itself, dependent on S2′ cleavage.

To avoid this misunderstanding, we have revised the relevant text (lines 73–77) as follows: "*Proteolytic cleavage at the S2' site is required for membrane fusion, as it promotes the extensive conformational rearrangements of the S2 subunit that enable its collapse into the HR1-HR2 six-helix bundle.*" The references suggested by the reviewer have been cited accordingly.

4. Lines 79-81: If the authors have evidence of cooperativity ("cooperative transitions") please include them in the manuscript.

We thank the reviewer for this helpful comment. We agree that the term "cooperative transitions" implies a mechanistic interpretation that is not directly supported by our data. Our cryo-EM analysis identifies distinct conformational states at different incubation times but does not directly assess whether the transitions between these states occur cooperatively. To avoid overinterpretation, we have replaced "cooperative transitions" with the more neutral phrase "*conformational transitions*" in the manuscript. The revised text more accurately reflects the scope of our structural observations.

5. Lines 95-98: Why choosing 24 and 72h which are very long time points compared to the time scale of membrane fusion? SARS-CoV-2 EFIC was discovered by using much shorter incubation times with ACE2 (PMID: 39889696). This reviewer can only assume that the authors decided to use extended incubation times as they did not even detect postfusion spikes after a 24h incubation with DPP4 (which argues against point #2).

Response: We thank the reviewer for this helpful comment. The choice of 24 h and 72 h as the principal time points was guided by the objective of capturing structurally informative conformational populations under our experimental conditions, rather than by the physiological timescale of membrane fusion. Our incubations were

performed using purified soluble MERS-CoV spike ectodomain and soluble DPP4 at 25 °C in pH 8.0 buffer, in the absence of membranes, proteases, or other host factors. These deliberately simplified conditions substantially slow receptor-induced conformational progression compared with viral entry in living cells. As a result, prolonged incubation was required to accumulate sufficient numbers of the less populated conformational states for high-resolution cryo-EM analysis.

To determine the appropriate incubation times, we first screened samples collected at different time points by negative-stain EM to monitor the overall progression of conformational changes. Based on these preliminary observations, we selected time points that captured distinct conformational populations for subsequent cryo-EM analysis. Cryo-EM datasets were collected after 1 h, 24 h, 72 h, and 120 h of incubation.

At 1 h, the sample was dominated by prefusion conformations, primarily the 1-up and 2-up states. At 24 h, the 2-up state predominated, and a very small population (~2%) of postfusion-like particles was already visible in the 2D class averages; however, these particles were too sparse and heterogeneous for high-resolution 3D reconstruction. By 72 h, the proportion of postfusion particles had increased sufficiently to enable high-resolution 3D reconstruction, while prefusion conformations were still present. At 120 h, the sample consisted almost exclusively of postfusion spikes, with no prefusion trimers detected. We have now included the 1 h and 120 h cryo-EM datasets in Fig 1， Figs S3 and S8, respectively.

These observations led us to select the 24 h and 72 h datasets for more detailed structural analysis, as they captured representative conformational populations before and after the emergence of abundant postfusion spikes.

6. Why is Fig1A showing a DPP4 dimer bound to two RBDs while the spike structures show a single RBD bound per DPP4 dimer?

Response: We thank the reviewer for this careful observation. We apologize that the original figure could lead to confusion.

In the spike trimer, each DPP4 dimer is modeled in association with a single RBD because density corresponding to a second RBD was not sufficiently resolved in the cryo-EM maps. This most likely reflects the limited local resolution and intrinsic flexibility of the receptor-bound RBD. In contrast, the isolated RBD–DPP4 reconstruction shows density consistent with a DPP4 dimer engaging two RBDs. However, as discussed in the revised manuscript, the particle population contributing to this reconstruction is likely heterogeneous and may include dissociated RBD–DPP4 complexes as well as intact spike trimers with highly flexible receptor-bound RBDs.

Therefore, the precise origin of this reconstruction cannot be unambiguously assigned. To avoid the confusion, we removed this from Figure 1.

7. FigS4 and S6 show that the cryoEM maps suffer from severe preferred orientation, limiting the quality of the maps and interpretations (except for the post-fusion map).

Response: We thank the reviewer for pointing out the preferred orientation issue. We agree that cryoEM maps (except for the postfusion map) exhibit noticeable preferred orientation, especially RBD-DPP4. For RBD-DPP4, we reprocessed the RBD-DPP4 to improve this situation.

The impact of preferred orientation differs among the reconstructed conformations. Despite the overall anisotropic particle distribution, the RBD-DPP4 and 3-down reconstructions reached high resolution without obvious density stretching or distortion, indicating that these two conformations are relatively less affected. For the 1-up, 2-up, and 3-up conformations, the map quality in the RBD and DPP4 regions is indeed lower. However, the key regions used for residue-level interaction analysis (e.g., RBD interfaces and CH helix key residues) still exhibit clear side-chain density. To support the residue-level analyses presented in the manuscript, we have added local-resolution maps and representative density for the key interface residues discussed in the text (Supplementary Fig S9). These regions display well-defined side-chain density, supporting the atomic modeling and the residue-level interactions described in the manuscript.

For structural regions that are more severely affected by preferred orientation or exhibit lower local resolution, we have revised the corresponding text throughout the manuscript to use more cautious language. Specifically, statements such as "demonstrates" have been replaced with more appropriate terms including "*suggests,*" "*is consistent with*," or "*may contribute to*," thereby avoiding overinterpretation of features that are not directly supported by the cryo-EM density (lines 202-231).

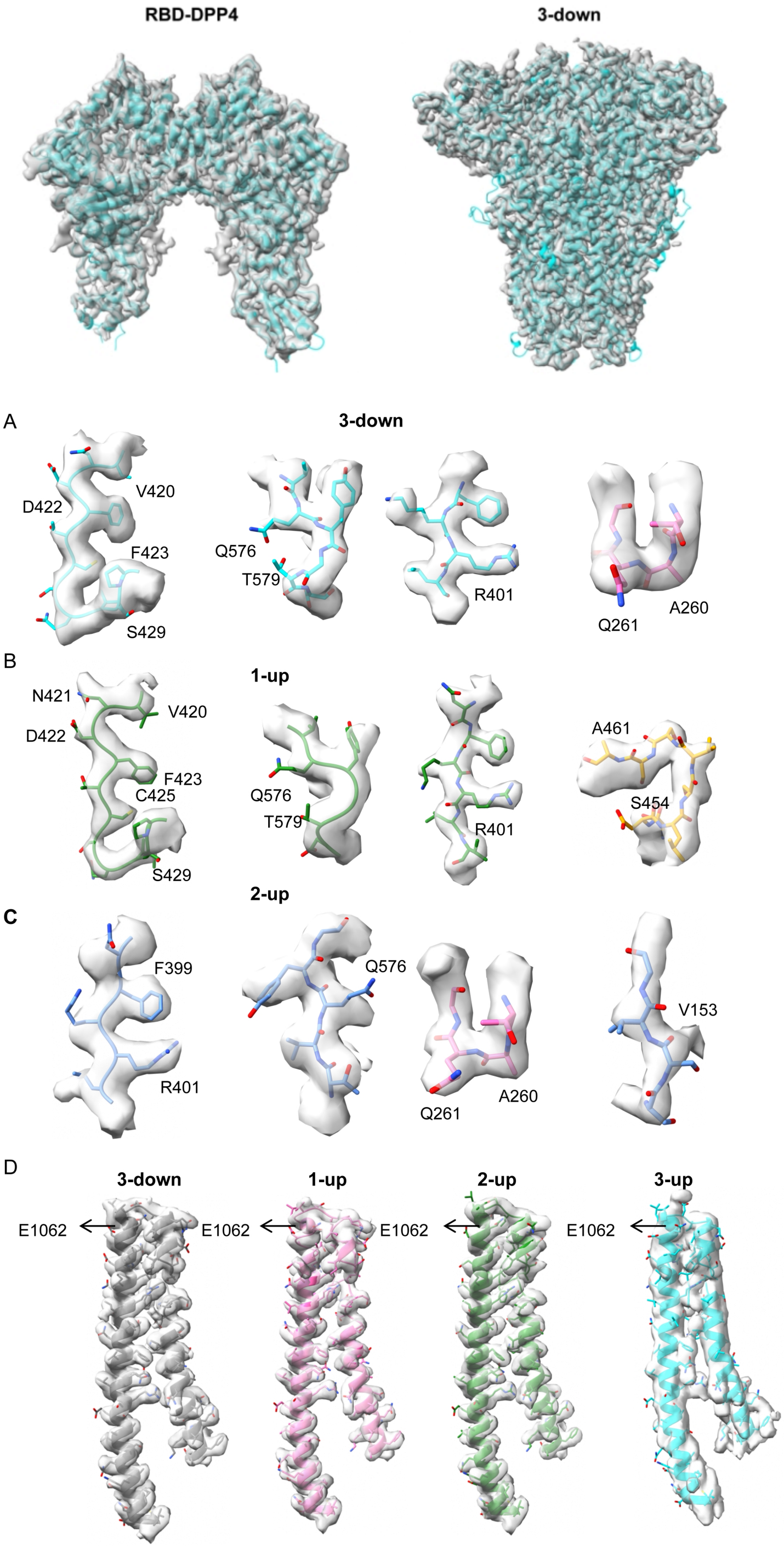

Figure S9. Local resolution density maps supporting the structural interpretations in Figure 3 and Figure 4.

(A) Representative cryo-EM density for key RBD interface residues from the 3-down conformation (Figure 3). (B) Representative density for key residues involved in the 1-up state at the RBD interface (Figure 3). (C) Representative density for key residues in the 2-up state at the RBD interface (Figure 3). (D) Density for the S2 core CH helix region and residue E1062 from Figure 4.

8. What conformational state is shown in the left-most part of Fig1D? The Pre-S2 label suggests the authors interpreted it as prefusion S2 subunit? This is a highly metastable state which is unexpected to be observed on its own. Moreover, no 2D averages, reconstruction workflow or FSC of a prefusion S2 subunit are shown in FigS5-S6.

Response: We thank the reviewer for raising this important point. We agree that the original label "Pre-S2" was overly specific and could imply an assignment that is not directly supported by our data. We therefore no longer refer to this density as a prefusion S2 subunit. Instead, we have renamed this reconstruction as a "**putative S2 intermediate**". We propose that it may represent an S2 subunit undergoing conformational rearrangement during the transition from the prefusion to the postfusion state. However, because this particle population is highly heterogeneous, only a low-resolution reconstruction (~6.6 Å) could be obtained, preventing an unambiguous structural assignment. We have therefore intentionally adopted more cautious terminology. The 2D class averages, FSC curve, and reconstruction workflow for this map have been included in Fig S5 and Fig S6. These additions allow readers to evaluate the quality of the reconstruction and the basis for our interpretation.

9. Lines 117-119: The observation of dissociated S1 trimers has previously been observed and should be cited (PMID: 28393837). Also, this argues against point #3 from the authors.

Response: We thank the reviewer for pointing out this important previous study. We have now cited the work (PMID: 28393837) in the revised manuscript.

Regarding the comment that this observation appears to argue against point #3, we apologize that our original wording may have caused confusion. As clarified in our response to Comment 3 and in the revised manuscript, we do not propose that S2′ cleavage directly causes or is required for S1 shedding. Rather, S2′ cleavage activates the fusion machinery by enabling the conformational rearrangements of the S2 subunit required for membrane fusion, whereas S1 shedding results from destabilization of the S1–S2 interface and is a distinct event.

The previous study (PMID: 28393837) demonstrated spontaneous S1 trimer dissociation in the absence of receptor, whereas our study examines conformational changes following DPP4 binding. We therefore consider these observations to be complementary. In our structures, receptor binding is accompanied by progressive

weakening of the S1–S2 interface and the appearance of dissociated S1 trimers, which is consistent with receptor engagement promoting S1 dissociation under our experimental conditions. We have revised the manuscript accordingly and cited the suggested reference.

10. How many times was the cryoEM classification repeated and how many biological replicates have the authors carried out to ensure the robustness of their interpretations?

Response: We thank the reviewer for raising this important question regarding the robustness of our interpretations. We address it from two aspects:

1) Cryo-EM classification: We have substantially expanded the Methods section to describe the data-processing workflow in detail. Briefly, all datasets were processed using RELION (v3.1) and cryoSPARC (v4.4.1). Following motion correction, CTF estimation, particle picking, and two rounds of 2D classification, particles were subjected to heterogeneous refinement in cryoSPARC. Each heterogeneous refinement class then underwent two independent rounds of 3D classification before non-uniform refinement. To assess potential bias introduced by Topaz particle picking, we also trained independent Topaz models using different conformational states as templates and repeated the particle-picking and classification procedure. Although the absolute proportions of individual classes changed slightly after reprocessing, the overall conformational distributions and their temporal trends remained unchanged. The revised particle proportions have been updated in the manuscript.

2) Biological replicates and additional time-point data: We acknowledge that independent biological replicates (independent protein expression, purification, and grid preparation) were not performed, and we have explicitly noted this as a limitation of the present study. To strengthen the robustness of our conclusions, we have included additional cryo-EM datasets collected after 1 h and 120 h of incubation under the same experimental conditions. These four datasets (1 h, 24 h, 72 h, and 120 h) show a consistent progression in conformational populations, from receptor-bound prefusion states at 1 h, to the appearance of the 3-up state at 24 h, abundant postfusion particles at 72 h, and predominantly postfusion particles at 120 h. Although these additional datasets do not replace independent biological replicates, they provide complementary evidence that supports the consistency of the structural trends reported in this study.

We have clarified these points in the revised Methods (lines 438–464) and Results (lines 102–122).

11. Lines 126-128: The MERS-CoV spike with three RBDs up has been previously observed and it should be cited (PMID: 28807998). This also applies to the claim made on lines 292-293.

Response: We thank the reviewer for the suggestion. As requested, we have added the citation (PMID: 28807998; Pallesen et al., 2017) at lines 162-169. In addition, we have revised the corresponding text to clarify that the previously reported 3up-RBDs state was observed in an engineered stabilized construct (S-2P) in the absence of DPP4 binding, whereas our study captures this state in the wild-type protein during DPP4s induced. These revisions are reflected in the manuscript (lines 162-169) as follows:"*Previous structural studies of the MERS-CoV S-DPP4 complex have been largely been confined to the isolated RBD. Cryo-EM analyses of the spike trimer have captured prefusion states with one, two, or three RBDs in the "up" conformation, but all of these were observed in an engineered prefusion-stabilized construct (S-2P) in the absence of DPP4 binding*[22] *. In contrast, the structure of the wild-type (WT) MERS-CoV spike ectodomain in complex with DPP4 has not previously been reported. One possible explanation is that receptor binding promotes conformational changes that reduce the stability of the prefusion spike.*"

12. Lines 149-152: This mechanism has been previously described and should be cited (PMID: 30712865).

Response: We thank the reviewer for the suggestion. The citation (PMID: 30712865) has been added at lines 184-187.

13. Lines 153-156: Where are the authors showing a single DPP4 dimer bridging two spike trimers? In any case, it has also been observed for SARS-CoV-2 and should be cited appropriately (PMID: 39146425).

Response: We thank the reviewer for the comment. Our data do not provide direct structural evidence that a single DPP4 dimer bridges two intact spike trimers. The direct observation from our study is that the isolated RBD–DPP4 reconstruction shows one DPP4 dimer simultaneously engaging two RBDs. Based on this observation, we speculate that similar DPP4-mediated crosslinking between intact spike trimers may occur on the cell surface, but this remains a hypothesis at present. As suggested, we have cited PMID: 39146425, which reports a similar phenomenon in SARS-CoV-2, and discuss this in Lines 389-394 as follows: "*Our isolated RBD-DPP4 reconstruction also revealed a DPP4 dimer simultaneously engaging two*

RBDs. Although our data do not provide direct evidence that a single DPP4 dimer bridges two intact spike trimers, they raise the possibility that receptor-mediated crosslinking could occur on the cell surface. If present in vivo, such interactions might influence spike organization or antibody accessibility and therefore warrant further investigation.”

14. Lines 176-195: it seems counter-intuitive to claim that the two RBDs up state is unstable when it is the most populated state after 24h (which again is a very long time relative to membrane fusion). The authors should also consider the metastability of S2 which will influence the overall trimer stability.
It seems unlikely that MERS-CoV would not transition through EFIC. It is more likely that the authors did not identify the right conditions to observe this intermediate.

Response: We thank the reviewer for their careful reading and constructive suggestions.

1. Conformational properties of the 2-up state.

We agree that describing the 2-up conformation as "unstable" was potentially misleading, particularly because it is the predominant prefusion state at 24 h. Our intention was not to imply that the 2-up state is intrinsically short-lived, but rather that it represents a conformational state with an increased propensity to progress to subsequent 3-up states under our experimental conditions. Consistent with this interpretation, the proportion of the 2-up state decreased from approximately 40% at 24 h to 12% at 72 h, whereas the 1-up state showed a comparatively smaller change (25% to 18%).

The structural analysis provides observations that are consistent with this interpretation. Compared with the 1-up conformation, the remaining down RBD in the 2-up state forms substantially fewer stabilizing hydrogen bonds, and the S2 core exhibits a measurable conformational displacement. We agree with the reviewer that the intrinsic metastability of the S2 subunit is also likely to contribute to the overall stability of the spike trimer, and we have incorporated this point into the revised manuscript.

2. Early fusion intermediate (E-FIC)

We agree that an E-FIC-like state may also exist during MERS-CoV spike activation. Our study does not exclude this possibility. As the reviewer suggests, the experimental conditions used here may not be optimal for stabilizing this transient intermediate. We have therefore revised the final paragraph of Discussion to state that the existence of an E-FIC-like state in MERS-CoV remains an open question that will

require future studies under more physiological conditions, as follows (lines 366-370): "*We did not capture an E-FIC analogous to that reported for SARS-CoV-2 under our experimental conditions (25 °C, soluble proteins, and no membrane environment), this does not exclude the existence of a similar intermediate in MERS-CoV, which may require different experimental conditions to stabilize*."

15. Lines 213-219: The data presented do not support the claims made, particularly the exertion of an "internal pressure on S1" or that "SARS-CoV-2 requires engagement of three ACE2 receptors to initiate fusion".

Response: We thank the reviewer for pointing this out. We agree that the original wording was too definitive and have revised the manuscript as follows:

1."Internal pressure on S1" has been changed to "*may exert an internal pushing force*", based on the observed outward drift of the S2 core and the weakened S1-S2 interface.

2."SARS-CoV-2 requires engagement of three ACE2 receptors to initiate fusion" has been revised to "*optimal fusion of SARS-CoV-2 may require engagement of up to three ACE2 receptors*", with citation of the relevant literature (Xing et al., 2025).

The corresponding revisions have been made in the main text (lines 248-255), as follows: "*These observations suggest that MERS-CoV and SARS-CoV-2 may employ different strategies for fusion activation. Our structural observations are consistent with a receptor-induced activation model for MERS-CoV, in which the DPP4 binding is accompanied by progressive outward displacement of the S2 core. This displacement may weaken interactions between S1 and S2, promoting S1 dissociation. By comparison, previous studies have suggested that efficient activation of the SARS-CoV-2 spike requires up to three ACE2 receptors8, thereby preventing premature activation by one or two receptors and ensuring that fusion occurs only under optimal conditions*". We appreciate the reviewer's rigorous reading.

16. Lines 226-231: the links between the undemonstrated SARS-CoV-2 spike stability and efficient transmission is unsubstantiated. Instead, the expression of ACE2 in the upper human airways is likely to participate in efficient SARS-CoV-2 transmission (PMID: 32526206). The poor transmission of MERS-CoV among humans is likely due to the predominant expression of DPP4 in the lower airways - MERS-CoV seems to transmit well among camels where DPP4 is expressed in the upper airways (PMID: 26889022, PMID: 25425139). Same for lines 321-324.

Response: We thank the reviewer for pointing this out. We agree that the direct link between SARS-CoV-2 spike stability and efficient airborne transmission is insufficiently supported. Accordingly, we have removed the speculative statements related to spike stability and transmission efficiency. We appreciate the reviewer's rigorous critique.

17. Lines 235-239: receptor-binding affinity would also participate here

Response: We thank the reviewer for the comment. We agree that receptor-binding affinity plays an important role in viral infection and transmission. The original text (lines 270-272) briefly mentions "including receptor binding affinity," but as our study focuses on structural mechanisms, we did not pursue an in- depth analysis of this aspect. We appreciate the reviewer's understanding.

18. Lines 246-249: What about the MHV post-fusion spike structure (PMID: 29073020)?

Response: We thank the reviewer for the suggestion. We have now included the postfusion S2 structure of MHV (PDB: 6B3O; Walls et al., 2017) in Figure 5D, alongside the postfusion S2 structures of MERS-CoV, SARS-CoV, and SARS-CoV-2 for direct comparison. The corresponding citation (PMID: 29073020) has been added in the main text (lines 285-288). The updated Figure 5D further supports the high conservation of the postfusion conformation among β-coronavirus S2 subunits. We appreciate the reviewer's valuable input.

19. Lines 269-271: Again the authors widely over-interpret their data

Response: We agree that the wording in lines 269–271 regarding the HR2 upstream linker as a "critical conformational switch" and "promising target" was overly definitive. The sentence has been revised as follows (lines 383-388): "*Despite these differences, the postfusion S2 architecture is highly conserved among MERS-CoV, SARS-CoV and SARS-CoV-2. In particular, the HR2 upstream linker undergoes a pronounced conformational rearrangement from a β-sheet to an extended loop. Together with its structural conservation across β-coronaviruses, this rearrangement suggests that the HR2 linker may represent a potential target for the development of broad-spectrum fusion inhibitors and neutralizing antibodies.*"

In addition, we have reviewed the entire manuscript and toned down other similar over-interpretations.

20. Lines 300-303: The authors claim that "The 2up-RBD population decrease from 47% on day one to 3% on day three, coinciding with the emergence of the 3up-RBD conformation, suggesting that the 2up-RBDs conformation can convert into the 3up-RBDs conformation." but no population with three RBDs up is detected at 72h.

Response: We thank the reviewer for pointing this out. We have revised the relevant description in the Results section (lines 138–148) to eliminate ambiguity and clarify the specific quantitative data. The revised text reads as follows: "*In contrast, analysis of the MERS-CoV S-DPP4 complex after 72 h of incubation revealed six distinct conformational states (Figure 1E), with a markedly different distribution from that observed at 24 h. Among intact S trimers, the proportion of the 1-up state decreased modestly from 25% to 18%, whereas the 2-up state declined more substantially from 40% to 12%. The 3-up state was no longer detected. In addition, a dissociated MERS-CoV S1 trimer was reconstructed to 5.85 Å resolution (Figure 1C and Figure S6), revealing a ring-like assembly composed of the NTD, RBD, SD1, and SD2 domains, with all three RBDs adopting the 'up' conformation. These observations suggest that the fully open (3-up) conformation is intrinsically unstable and prone to S1 dissociation, which may account for the absence of the 3-up state at 72 h.*"

In addition, the Discussion section (lines 350–360) has been revised accordingly, as follows: "*Previous studies have shown that the WT MERS-CoV spike in the absence of receptor can adopt multiple prefusion conformations: 3-down, 1-up, and 2-up RBDs. Our data further show that prolonged incubation with DPP4 results in the appearance of the fully open 3-up state, followed by S1 dissociation. Receptor binding is accompanied by conformational changes in the S2 core and a progressive reduction in stabilizing interactions surrounding the remaining down RBD, which may facilitate its transition to the up conformation. When all three RBDs adopt the "up" conformation, the interactions between S1 and S2 are weakened, facilitating the dissociation of S1 trimer ring. The S2 subunit subsequently refolds into the characteristic six-helix bundle, drawing the viral and host membranes together to complete membrane fusion (Figure 6)*". We appreciate the reviewer's rigorous assessment.

21. Lines 314-316: A 4Å shift does not qualify as "pronounced" based on the magnitude of the spike rearrangements observed during membrane fusion.

Response: We thank the reviewer for pointing this out. We have removed the word "pronounced" and revised the relevant wording accordingly. The revision has been

made in lines 373–376.

Reviewer #3: 1. The current wording in the title, abstract, and discussion is somewhat stronger than necessary. With two endpoint comparisons rather than a dense temporal series, “time-resolved” feels overstated. Likewise, while the data support a receptor-associated or receptor-coupled activation pathway, some formulations read more definitively than the experimental design warrants. I encourage moderating the language throughout to improve precision. The comparison between the 24 h and 72 h samples is informative, and the particle distributions clearly differ, but with only two relatively distant endpoints the manuscript should avoid implying a continuously resolved temporal pathway. It would help to clarify why these two time points were chosen and what conclusions can and cannot be drawn from them. As written, this is a convincing time-dependent comparison, but not a fully time-resolved kinetic analysis.

Response: We thank the reviewer for these thoughtful comments. We have carefully revised the manuscript accordingly to improve the precision of our wording and to more clearly define the scope of the conclusions supported by the data.

(1) We agree that the term “time-resolved” may imply observation of continuous progression of spike activation. Since our study compares structural populations obtained after defined incubation periods rather than capturing continuous kinetic transitions, we have replaced "time-resolved" with "time-course" (or "analysis at different incubation times") throughout the manuscript to more accurately reflect the experimental design. The title now reads: “*Receptor-induced activation of MERS-CoV spike revealed by time-course cryo-EM*”.

(2) Moderation of mechanistic language: Throughout the manuscript, we have revised statements that could be interpreted as implying definitive mechanisms. For example, terms such as "demonstrates" have been replaced with "suggests" or "is consistent with" where appropriate, and mechanistic interpretations are now presented as hypotheses supported by the structural observations rather than as established conclusions. We have also introduced qualifiers such as "may," "could," "likely," and "appears to" where appropriate.

(3) Clarification of the rationale for selecting the 24 h and 72 h time points. To determine informative time points for structural analysis, we first examined samples collected after different incubation periods by negative-stain EM and subsequently collected cryo-EM data at 1 h, 24 h, 72 h, and 120 h. At 1 h, only prefusion conformations (primarily the 1-up and 2-up states) were observed. At 24 h, all four prefusion conformations were present, together with a very small population (~2%) of

postfusion-like particles that was insufficient for high-resolution reconstruction. By 72 h, postfusion particles had increased to approximately 24%, enabling a 3.23 Å reconstruction while prefusion conformations were still retained. At 120 h, the sample consisted almost exclusively of postfusion spikes. We therefore selected the 24 h and 72 h datasets for detailed description because they represent distinct conformational populations before and after the emergence of abundant postfusion particles. The 1 h and 120 h cryo-EM datasets have now been included in the Supplementary Information (Figs. S3 and S8).

(4) Clarification of the scope of the conclusions: We have added a statement (lines 343–349) to the Discussion, as follows: "*Although our analysis is based primarily on two incubation time points (24 h and 72 h), with additional observations at 1 h and 120 h, the structures reveal distinct conformational populations that outline a plausible pathway of receptor-induced spike activation. Because these datasets represent discrete structural snapshots rather than continuous kinetic measurements, our conclusions are limited to comparisons of the conformational states enriched under the experimental conditions used.*". We believe this clarification more accurately reflects both the strengths and the limitations of our experimental design.

We believe these revisions have substantially improved the precision of the manuscript and thank the reviewer for helping us present our conclusions in a more balanced and accurate manner.

2. In the sections comparing S1–S2 interfaces and interaction networks, the level of detail in describing specific residue-level interactions (particularly hydrogen bonds) appears stronger than warranted given the map resolutions. The structural trends are interesting and plausible, but the wording often implies a level of certainty that may not be justified across all regions. I recommend using more cautious language (e.g., "is consistent with," "suggests," "supports") unless the local map quality clearly supports residue-level interpretation. This is particularly relevant where interaction networks are used to explain population shifts between conformations. The same point applies to the interpretation of Fig.4, where mechanistic language should be better aligned with the resolution and variability of the maps.

Response: We thank the reviewer for this important suggestion. We agree that residue-level interpretations should be presented cautiously, particularly in regions where the local map quality does not fully support definitive assignment of specific interactions. We have therefore revised the manuscript to better align our interpretations with the resolution of the cryo-EM maps.

For Figure 2: We have substantially moderated the description of the S1–S2

interface. Rather than emphasizing individual hydrogen bonds, the revised text focuses on the overall changes in interfacial contacts and buried surface area during the conformational transitions. Statements implying definitive mechanisms have been replaced with more cautious wording, such as *"may contribute to," "is consistent with,"* and *"suggests."* These revisions are reflected in lines 175-187.

For Figure 3 and Figure 4: We have included new Supplementary Figure S9 showing local-resolution density maps for key residues (e.g., RBD interface residues R401/Q576 in Figure 3, and CH helix residue E1062 in Figure 4A), demonstrating relatively clear side-chain density in these regions. For these figures, we retain cautious wording such as “is supported by” and “is consistent with,” while still avoiding over-definitive language such as “definitively proves”. These revisions are reflected in lines 202–231.

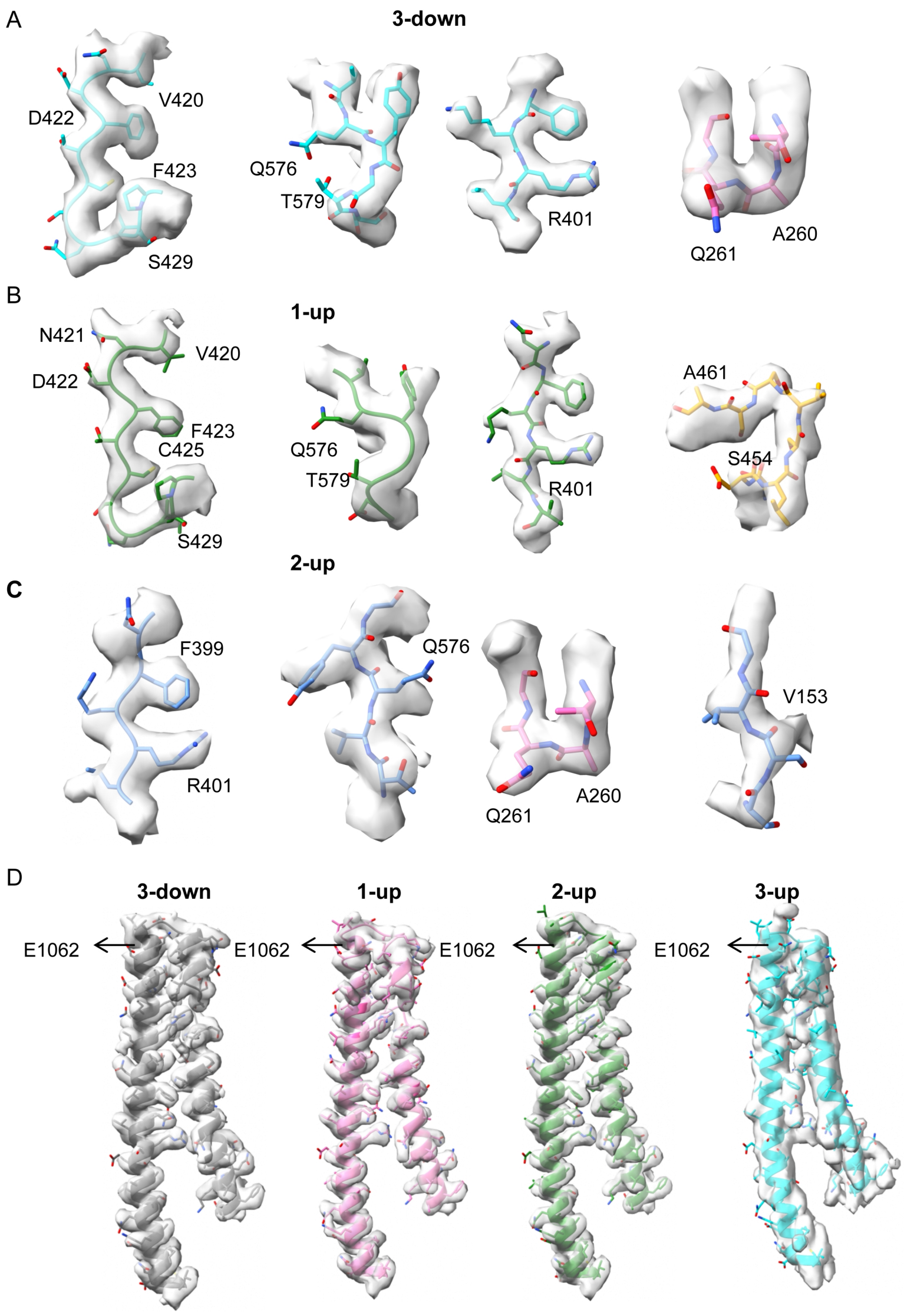

Figure S9. Local resolution density maps supporting the structural interpretations in Figure 3 and Figure 4.

(A) Representative cryo‐ EM density for key RBD interface residues from the 3-down conformation (Figure 3). (B) Representative density for key residues involved in the 1-up state at the RBD interface (Figure 3). (C) Representative density for key residues in the 2-up state at the RBD interface (Figure 3). (D) Density for the S2 core CH helix region and residue E1062 from Figure 4.

Overall, we have reviewed the manuscript and replaced overly definitive wording with more appropriately qualified language wherever residue-level interactions or mechanistic interpretations extend beyond the direct support provided by the cryo-EM maps.

3. The postfusion S2 structure is an important strength of the manuscript, and the rearrangement of the linker upstream of HR2 is clearly interesting. However, describing this region as a “critical conformational switch” or as an established therapeutic target goes beyond what is directly demonstrated structurally. In the absence of functional validation, these claims should be softened or explicitly framed as hypotheses. Even limited functional validation would strengthen this aspect, but careful revision of the wording should be sufficient.

We thank the reviewer for this constructive comment. We agree that, in the absence of direct functional validation, the original wording regarding the HR2 upstream linker was stronger than warranted by the structural data. We have therefore revised the manuscript to present these interpretations more cautiously.

We revised "critical conformational switch" to "may act as a conformational switch", and "established therapeutic target" to " a potential target for future investigation." We have also introduced qualifiers such as "may," "could," “suggesting that” and “could potentially” where appropriate, so that the discussion is clearly framed as a structural interpretation rather than a functionally established mechanism.

In addition, a recent study reported that a hydrophobic “lock” formed by residue F1148 in the HR2 upstream linker of SARS-CoV-2 was critical for six-helix bundle formation and membrane fusion, with mutations at this residue impairing fusion function. Structural comparison of the postfusion S2 subunits reveals that MERS-CoV F1231 corresponds to SARS-CoV-2 F1148 and resides in a structurally equivalent hydrophobic cavity (Fig. S10). This observation suggests that a similar hydrophobic lock may contribute to the refolding of the MERS-CoV S2 subunit. This finding further supports the potential functional importance of the HR2-linker region across β-coronaviruses.

We incorporated this analysis into the revised manuscript (Lines 311-322) ,as

follows: "*These observations suggest that stabilizing the prefusion conformation of the HR2 upstream linker, or preventing its rearrangement, may interfere with membrane fusion, highlighting this region as a potential target for the development of broad-spectrum antivirals and neutralizing antibodies. A recent study identified a hydrophobic "lock" centered on residue F1148 within the SARS-CoV-2 HR2 upstream linker that is required for efficient six-helix bundle assembly and membrane fusion, as mutations at this site impair fusion activity*[31]*. Structural comparison of the postfusion S2 subunits revealed that MERS-CoV F1231 corresponds to SARS-CoV-2 F1148 and resides in a structurally equivalent hydrophobic cavity (Fig. S10). This observation raises the possibility that a similar hydrophobic lock contributes to the refolding of the MERS-CoV S2 subunit. These observations are consistent with a conserved functional role of the HR2-linker region across β-coronaviruses.*"

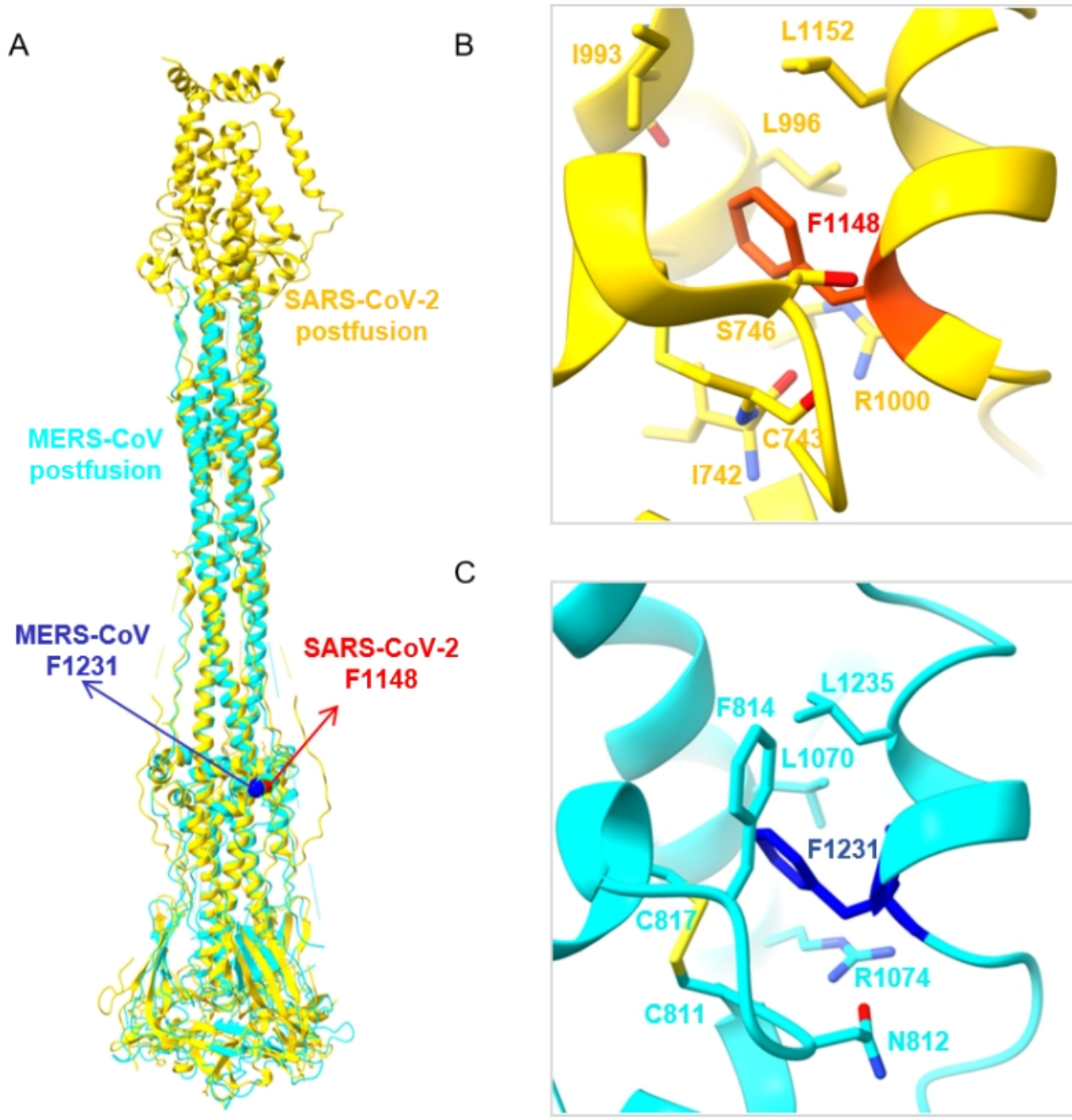

Figure S10. Structural comparison of the hydrophobic "lock" in the HR2 upstream linker between SARS-CoV-2 and MERS-CoV postfusion structures.

(A) Superimposed postfusion S2 subunits of SARS-CoV-2 (yellow, PDB: 8FDW) and MERS-CoV (cyan). (B) Detailed view of SARS-CoV-2 F1148 (red) embedded in the hydrophobic cavity formed by surrounding residues. (C) Detailed view of MERS-CoV F1231 (blue) occupying a structurally equivalent hydrophobic cavity.

4.In Fig.S3 (notably the RBD–DPP4 and 3down-RBD panels) and Fig.S5 (postfusion), the local-resolution density maps do not appear fully consistent with the corresponding 3D reconstructions. In some instances this may reflect differences in

thresholding, sharpening, or display parameters, but in others the presentation is difficult to reconcile. Harmonizing map display and clearly stating how maps were rendered need to be done.

We thank the reviewer for pointing out this issue. We have optimized the data processing workflow figures and now include show only the local-resolution density maps in the supplementary materials, shown as originally output by cryoSPARC without additional post-processing. To ensure consistency, the display parameters have been harmonized across all supplementary figures. We have also clarified the rendering procedure in the figure legends by adding the following statement: "All density maps were rendered in UCSF ChimeraX. The final cryo-EM maps are colored according to local resolution estimated by cryoSPARC with the Fourier shell correlation (FSC) = 0.143 criterion."

We appreciate the reviewer's help in improving the clarity and standardization of our data presentation.

Part III – Minor Issues: Editorial and Data Presentation Modifications

Please use this section for editorial suggestions as well as relatively minor modifications of existing data that would enhance clarity.

Reviewer #1: (No Response)

Reviewer #2: (No Response)

Reviewer #3: 1. The order of supplementary figure citation is not fully consistent throughout the manuscript. The sequence of citation should be standardized to improve readability.

We thank the reviewer for pointing out this issue. We have carefully checked and standardized the citations to supplementary figures throughout the manuscript to ensure that they appear sequentially (S1–S11).

2. The manuscript would benefit from careful English editing for grammar, syntax, and style. While generally understandable, there are multiple small issues that affect readability.

The manuscript has been carefully throughout to improve the grammar, syntax, and overall readability. We have also revised a number of sentences for clarity and consistency to ensure that the text more accurately conveys the intended scientific meaning.

3. There is an inconsistency in the author contributions section: “M.L.” is listed, but this author does not appear in the author list. I assume this is a typo and likely corresponds to Zhimin Liu.

Yes, “M.L.” refers to Zhimin Liu. We have revised it to “ Z.L.”.

4. Nzomenclature is inconsistent across the text and figures (e.g., 1up / 1-up / one-up; 3down / 3-down; postfusion / postfusion). This should be unified throughout for clarity and consistency.

We have unified the terminology as “1-up”, “2-up”, “3-up”, “3-down”, and “postfusion” throughout the text, figures, and supplementary materials.

pp6 LL109: “As contrast” → should read “In contrast”.
pp7 LL132–134: “multiple RBD-up intermediate complexed with DPP4” should be “multiple RBD-up intermediates complexed with DPP4”.
pp9 LL170–175: Several grammatical issues in this paragraph (e.g., “Together, these interactions locking RBDA…” should be “lock RBDA”).
pp11 LL232–233: “physical foundational” should be “physical foundation”.
pp16 LL357–358: “Plasmid is transfected…” should be “The plasmid was transfected…”
pp16 LL360: “buffer solution” redundant; “buffer” sufficient.
pp17 LL372–374: “A nominal magnification…” remove “A” or correct sentence structure.
pp17 LL380–381: “reported resolution is based…” missing article “The reported resolution…”.

We thank the reviewer for carefully identifying these language errors. The suggested corrections have been incorporated into the revised manuscript.
