# Supplementary material for: Receptor-induced activation of MERS-CoV spike revealed by time-course cryo-EM
Source: PLoS Pathog. 2026 Oct 8;22(10):e1014545. doi: 10.1371/journal.ppat.1014545 (PMC13648974; doi:10.1371/journal.ppat.1014545)
Supplement: Peer review history

# Peer review: Decision Letter 0

**DOI:** 10.1371/journal.ppat.1014545.r001

**Authors:** Alexander Gorbalenya, Nuria Verdaguer

7 Apr 2026

Receptor-triggered fusion mechanism of MERS-CoV revealed by time-resolved cryo-EM

PLOS Pathogens

Dear Dr. Sun,

Thank you for submitting your manuscript to PLOS Pathogens. After careful consideration, we feel that it has merit but does not fully meet PLOS Pathogens's publication criteria as it currently stands. Therefore, we invite you to submit a revised version of the manuscript that addresses the points raised during the review process.

Please submit your revised manuscript by 60 days. If you will need more time than this to complete your revisions, please reply to this message or contact the journal office at plospathogens@plos.org. When you're ready to submit your revision, log on to https://www.editorialmanager.com/ppathogens/ and select the 'Submissions Needing Revision' folder to locate your manuscript file.

Please include the following items when submitting your revised manuscript:

* A letter that responds to each point raised by the editor and reviewer(s). You should upload this letter as a separate file labeled 'Response to Reviewers'. This file does not need to include responses to any formatting updates and technical items listed in the 'Journal Requirements' section below.

* A marked-up copy of your manuscript that highlights changes made to the original version. You should upload this as a separate file labeled 'Revised Manuscript with Track Changes'.

* An unmarked version of your revised paper without tracked changes. You should upload this as a separate file labeled 'Manuscript'.

If you would like to make changes to your financial disclosure, competing interests statement, or data availability statement, please make these updates within the submission form at the time of resubmission. Guidelines for resubmitting your figure files are available below the reviewer comments at the end of this letter.

We look forward to receiving your revised manuscript.

Kind regards,

Nuria Verdaguer

Academic Editor

PLOS Pathogens

Alexander Gorbalenya

Section Editor

PLOS Pathogens

Editor-in-Chief

PLOS Pathogens

orcid.org/0000-0003-2946-9497

Michael Malim

Editor-in-Chief

PLOS Pathogens

orcid.org/0000-0002-7699-2064

**Additional Editor Comments:**

As you will see from the reviewers reports, several important concerns have been raised.  In particular, all three reviewers agree that the manuscript overstates its conclusions and employs mechanistic language that is not fully supported by the data. The characterization of the study as “time-resolved” (including the **title** ) is considered misleading, as it relies on only two widely separated time points (24 and 72 hours) and does not capture true kinetics or continuous dynamics. There is also a consistent concern regarding the lack of functional or experimental validation to support key claims, including those related to stability, activation mechanisms, and biological relevance.

In addition, the robustness and reproducibility of the structural analysis are questioned in several instances. The authors are asked to provide clearer methodological details, including validation of classification approaches and evidence of reproducibility. Furthermore, the manuscript does not sufficiently engage with the existing literature, as relevant studies are sometimes not cited and previously reported findings are presented as novel. Finally, the resolution of the maps does not support the level of detail used to describe specific interactions, particularly hydrogen bonds, and these interpretations should be presented more cautiously.

**Journal Requirements:**

1) Please ensure that the CRediT author contributions listed for every co-author are completed accurately and in full.

At this stage, the following Authors/Authors require contributions: Yajia Wang, Xiyuan Pan, Yuxuan Jiang, Zhimin Liu, Yinong Qiu, Zhenguo Chen, and Lei Sun. Please ensure that the full contributions of each author are acknowledged in the "Add/Edit/Remove Authors" section of our submission form.

The list of CRediT author contributions may be found here: https://journals.plos.org/plospathogens/s/authorship#loc-author-contributions

2) We ask that a manuscript source file is provided at Revision. Please upload your manuscript file as a .doc, .docx, .rtf or .tex. If you are providing a .tex file, please upload it under the item type u2018LaTeX Source Fileu2019 and leave your .pdf version as the item type u2018Manuscriptu2019.

3) Please upload all main figures as separate Figure files in .tif or .eps format. For more information about how to convert and format your figure files please see our guidelines:

https://journals.plos.org/plospathogens/s/figures

4) We have noticed that you have uploaded Supporting Information files, but you have not included a list of legends. Please add a full list of legends for your Supporting Information files after the references list.

5) Please amend your detailed Financial Disclosure statement. This is published with the article. It must therefore be completed in full sentences and contain the exact wording you wish to be published.

State what role the funders took in the study. If the funders had no role in your study, please state: "The funders had no role in study design, data collection and analysis, decision to publish, or preparation of the manuscript.".

If you did not receive any funding for this study, please simply state: u201cThe authors received no specific funding for this work.u201d

**Reviewers' Comments:**

Reviewer's Responses to Questions

**Part I - Summary**

Please use this section to discuss strengths/weaknesses of study, novelty/significance, general execution and scholarship.

Reviewer #1: In this article, Wang and colleagues study the mechanism of DPP4-mediated activation of the MERS spike using cryo-EM at two different time points, 24 and 72 hours. They report structures of the prefusion (3 RBDs down), intermediate (3, 2, and 1 RBD-up), and postfusion states, allowing them to reconstruct a trajectory of spike activation and to observe how receptor binding weakens the interactions between S1 and S2. The results presented in this article are consistent with what has been previously described for other coronaviruses, help improve our understanding of MERS entry mechanisms, and may have implications for the development of antiviral therapies. I only have a few comments that I hope will help improve the manuscript.

Reviewer #2: In this manuscript, Wang et al describe cryoEM structures of the MERS-CoV spike incubated for various amounts of times with the DPP4 receptor. Although the premise of the work is interesting, the manuscript provides limited new insights and is not suitable as is for publication.

Reviewer #3: This study by Wang et al. is an interesting structural manuscript that substantially advances our understanding of MERS-CoV spike activation. The authors capture receptor-bound 1-up, 2-up, and 3-up prefusion states and determine a postfusion S2 structure. Overall, I found the study novel, informative, and appropriate for the readership of PLOS Pathogens. My main reservation is not the quality or interest of the dataset, but rather that some of the mechanistic language is stronger than necessary. In particular, terms such as “time-resolved,” “direct receptor-triggered fusion mechanism,” and some of the more detailed interpretations of residue-level interactions would benefit from softening. That said, these issues do not undermine the core value of the work. I would therefore support publication after revision.

**********

**Part II – Major Issues: Key Experiments Required for Acceptance**

Please use this section to detail the key new experiments or modifications of existing experiments that should be absolutely required to validate study conclusions.

Generally, there should be no more than 3 such required experiments or major modifications for a "Major Revision" recommendation. If more than 3 experiments are necessary to validate the study conclusions, then you are encouraged to recommend "Reject".

Reviewer #1: 1. The authors mention that they use “time-resolved” cryo-EM, but they only show structures of discrete states rather than continuous dynamics. What is the rate (speed) of the reaction? They analyze structures after 24 and 72 hours of incubation with a slight molar excess of receptor, which suggests slow activation kinetics. However, MERS infection experiments (for example, Coleman and Frieman, 2015) show replication products within 12 hours. How can these two observations be reconciled? The authors should include functional data showing the kinetics of receptor-spike interaction, for example using BLI, or data showing that postfusion forms only appear after days of incubation.

2. What is the distribution of spike conformations at 24 h and 72 h in the absence of receptor? And what is the distribution in the presence of receptor at 1 h?

3. One possibility is that the observed differences are due to artifacts related to sample preparation or cryo-EM data processing. How reproducible are these results?

4. How robust is the particle distribution analysis shown in Figure 1? The authors perform 3D classification using relion, would they obtain the same distribution using cryosparc? To select particles, they train a topaz model to identify the intermediate states. How dependent are the results on this topaz model? How many 3D classes do they use in relion during classification, and how does the final result depend on the number of classes used? The authors should be more explicit about these details in the Materials and Methods section.

5. The authors discuss structural differences relative to the SARS-CoV-2 spike. How do the kinetics of activation compare between the two? Are the infection kinetics of the two viruses also different?

Reviewer #2: 1. The abstract is misleading as it states that the manuscript describes structures obtained though “time-resolved series” including an “intermediate”. No such intermediate is described.

2. The authors claim that the SARS-CoV-2 spike is highly stable as compared to the MERS-CoV spike but no experimental measurements are provided to support this claim

3. Lines 67-70: the authors seem to suggest that processing of MERS-CoV S2’ leads to S1 shedding. What mechanism do the authors envision would lead to this? Why is S1 shedding requiring S2’ proteolytic cleavage given that S is already cleaved at S1/S2. The consensus in the field is that S2’ cleavage promotes membrane fusion (e.g. PMID: 27791014, PMID: 25288733).

4. Lines 79-81: If the authors have evidence of cooperativity (“cooperative transitions”) please include them in the manuscript.

5. Lines 95-98: Why choosing 24 and 72h which are very long time points compared to the time scale of membrane fusion? SARS-CoV-2 EFIC was discovered by using much shorter incubation times with ACE2 (PMID: 39889696). This reviewer can only assume that the authors decided to use extended incubation times as they did not even detect postfusion spikes after a 24h incubation with DPP4 (which argues against point #2).

6. Why is Fig1A showing a DPP4 dimer bound to two RBDs while the spike structures show a single RBD bound per DPP4 dimer?

7. FigS4 and S6 show that the cryoEM maps suffer from severe preferred orientation, limiting the quality of the maps and interpretations (except for the post-fusion map).

8. What conformational state is shown in the left-most part of Fig2D? The Pre-S2 label suggests the authors interpreted it as prefusion S2 subunit? This is a highly metastable state which is unexpected to be observed on its own. Moreover, no 2D averages, reconstruction workflow or FSC of a prefusion S2 subunit are shown in FigS5-S6.

9. Lines 117-119: The observation of dissociated S1 trimers has previously been observed and should be cited (PMID: 28393837). Also, this argues against point #3 from the authors.

10. How many times was the cryoEM classification repeated and how many biological replicates have the authors carried out to ensure the robustness of their interpretations?

11. Lines 126-128: The MERS-CoV spike with three RBDs up has been previously observed and it should be cited (PMID: 28807998). This also applies to the claim made on lines 292-293.

12. Lines 149-152: This mechanism has been previously described and should be cited (PMID: 30712865).

13. Lines 153-156: Where are the authors showing a single DPP4 dimer bridging two spike trimers? In any case, it has also been observed for SARS-CoV-2 and should be cited appropriately (PMID: 39146425).

14. Lines 176-195: it seems counter-intuitive to claim that the two RBDs up state is unstable when it is the most populated state after 24h (which again is a very long time relative to membrane fusion). The authors should also consider the metastability of S2 which will influence the overall trimer stability.

It seems unlikely that MERS-CoV would not transition through EFIC. It is more likely that the authors did not identify the right conditions to observe this intermediate.

15. Lines 213-219: The data presented do not support the claims made, particularly the exertion of an “internal pressure on S1” or that “SARS-CoV-2 requires engagement of three ACE2 receptors to initiate fusion”.

16. Lines 226-231: the links between the undemonstrated SARS-CoV-2 spike stability and efficient transmission is unsubstantiated. Instead, the expression of ACE2 in the upper human airways is likely to participate in efficient SARS-CoV-2 transmission (PMID: 32526206). The poor transmission of MERS-CoV among humans is likely due to the predominant expression of DPP4 in the lower airways - MERS-CoV seems to transmit well among camels where DPP4 is expressed in the upper airways (PMID: 26889022, PMID: 25425139). Same for lines 321-324.

17. Lines 235-239: receptor-binding affinity would also participate here

18. Lines 246-249: What about the MHV post-fusion spike structure (PMID: 29073020)?

19. Lines 269-271: Again the authors widely over-interpret their data

20. Lines 300-303: The authors claim that “The 2up-RBD population decrease from 47% on day one to 3% on day three, coinciding with the emergence of the 3up-RBD conformation, suggesting that the 2up-RBDs conformation can convert into the 3up-RBDs conformation.” but no population with three RBDs up is detected at 72h.

21. Lines 314-316: A 4Å shift does not qualify as “pronounced” based on the magnitude of the spike rearrangements observed during membrane fusion.

Reviewer #3: 1. The current wording in the title, abstract, and discussion is somewhat stronger than necessary. With two endpoint comparisons rather than a dense temporal series, “time-resolved” feels overstated. Likewise, while the data support a receptor-associated or receptor-coupled activation pathway, some formulations read more definitively than the experimental design warrants. I encourage moderating the language throughout to improve precision. The comparison between the 24 h and 72 h samples is informative, and the particle distributions clearly differ, but with only two relatively distant endpoints the manuscript should avoid implying a continuously resolved temporal pathway. It would help to clarify why these two time points were chosen and what conclusions can and cannot be drawn from them. As written, this is a convincing time-dependent comparison, but not a fully time-resolved kinetic analysis.

2. In the sections comparing S1–S2 interfaces and interaction networks, the level of detail in describing specific residue-level interactions (particularly hydrogen bonds) appears stronger than warranted given the map resolutions. The structural trends are interesting and plausible, but the wording often implies a level of certainty that may not be justified across all regions. I recommend using more cautious language (e.g., “is consistent with,” “suggests,” “supports”) unless the local map quality clearly supports residue-level interpretation. This is particularly relevant where interaction networks are used to explain population shifts between conformations. The same point applies to the interpretation of Fig. 4, where mechanistic language should be better aligned with the resolution and variability of the maps.

3. The postfusion S2 structure is an important strength of the manuscript, and the rearrangement of the linker upstream of HR2 is clearly interesting. However, describing this region as a “critical conformational switch” or as an established therapeutic target goes beyond what is directly demonstrated structurally. In the absence of functional validation, these claims should be softened or explicitly framed as hypotheses. Even limited functional validation would strengthen this aspect, but careful revision of the wording should be sufficient.

4.In Fig. S3 (notably the RBD–DPP4 and 3down-RBD panels) and Fig. S5 (postfusion), the local-resolution density maps do not appear fully consistent with the corresponding 3D reconstructions. In some instances this may reflect differences in thresholding, sharpening, or display parameters, but in others the presentation is difficult to reconcile. Harmonizing map display and clearly stating how maps were rendered need to be done.

**********

**Part III – Minor Issues: Editorial and Data Presentation Modifications**

Please use this section for editorial suggestions as well as relatively minor modifications of existing data that would enhance clarity.

Reviewer #1: (No Response)

Reviewer #2: (No Response)

Reviewer #3: 1. The order of supplementary figure citation is not fully consistent throughout the manuscript. The sequence of citation should be standardized to improve readability.

2. The manuscript would benefit from careful English editing for grammar, syntax, and style. While generally understandable, there are multiple small issues that affect readability.

3. There is an inconsistency in the author contributions section: “M.L.” is listed, but this author does not appear in the author list. I assume this is a typo and likely corresponds to Zhimin Liu.

4. Nzomenclature is inconsistent across the text and figures (e.g., 1up / 1-up / one-up; 3down / 3-down; post-fusion / postfusion). This should be unified throughout for clarity and consistency.

pp6 LL109: “As contrast” → should read “In contrast”.

pp7 LL132–134: “multiple RBD-up intermediate complexed with DPP4” should be “multiple RBD-up intermediates complexed with DPP4”.

pp9 LL170–175: Several grammatical issues in this paragraph (e.g., “Together, these interactions locking RBDA…” should be “lock RBDA”).

pp11 LL232–233: “physical foundational” should be “physical foundation”.

pp16 LL357–358: “Plasmid is transfected…” should be “The plasmid was transfected…”

pp16 LL360: “buffer solution” redundant; “buffer” sufficient.

pp17 LL372–374: “A nominal magnification…” remove “A” or correct sentence structure.

pp17 LL380–381: “reported resolution is based…” missing article “The reported resolution…”.

**********

PLOS authors have the option to publish the peer review history of their article (what does this mean?). If published, this will include your full peer review and any attached files.

If you choose “no”, your identity will remain anonymous but your review may still be made public.

**Do you want your identity to be public for this peer review?**  For information about this choice, including consent withdrawal, please see our Privacy Policy.

Reviewer #1: No

Reviewer #2: No

Reviewer #3: No

[NOTE: If reviewer comments were submitted as an attachment file, they will be attached to this email and accessible via the submission site. Please log into your account, locate the manuscript record, and check for the action link "View Attachments". If this link does not appear, there are no attachment files.]

**Figure resubmission:**

**Reproducibility:**

?>

# Author comment: Author response to Decision Letter 1

**DOI:** 10.1371/journal.ppat.1014545.r002

16 Jul 2026

Attachment Submitted filename: 9-Response to Reviewers-260714.pdf

# Peer review: Decision Letter 1

**DOI:** 10.1371/journal.ppat.1014545.r003

**Authors:** Alexander Gorbalenya, Nuria Verdaguer

12 Aug 2026

Dear Prof. Lei Sun,

We are pleased to inform you that your manuscript 'Receptor-induced activation of MERS-CoV spike revealed by time-course cryo-EM.' has been provisionally accepted for publication in PLOS Pathogens.

Before your manuscript can be formally accepted you will need to complete some formatting changes, which you will receive in a follow up email. A member of our team will be in touch with a set of requests.

Please note that your manuscript will not be scheduled for publication until you have made the required changes, so a swift response is appreciated.

IMPORTANT: The editorial review process is now complete. PLOS will only permit corrections to spelling, formatting or significant scientific errors from this point onwards. Requests for major changes, or any which affect the scientific understanding of your work, will cause delays to the publication date of your manuscript.

Should you, your institution's press office or the journal office choose to press release your paper, you will automatically be opted out of early publication. We ask that you notify us now if you or your institution is planning to press release the article. All press must be co-ordinated with PLOS.

Thank you again for supporting Open Access publishing; we are looking forward to publishing your work in PLOS Pathogens.

Best regards,

Nuria Verdaguer

Academic Editor

PLOS Pathogens

Alexander Gorbalenya

Section Editor

PLOS Pathogens

Sumita Bhaduri-McIntosh

Editor-in-Chief

PLOS Pathogens

orcid.org/0000-0003-2946-9497

Michael Malim

Editor-in-Chief

PLOS Pathogens

orcid.org/0000-0002-7699-2064

***********************************************************

Editor Comments:

The authors have properly addressed all the criticisms and suggestions from the reviewers

Reviewer Comments (if any, and for reference):

Reviewer's Responses to Questions

**Part I - Summary**

Please use this section to discuss strengths/weaknesses of study, novelty/significance, general execution and scholarship.

Reviewer #1: The authors have responded to all of my comments and I have no further comments to add. I congratulate them on their work.

**********

**Part II – Major Issues: Key Experiments Required for Acceptance**

Please use this section to detail the key new experiments or modifications of existing experiments that should be absolutely required to validate study conclusions.

Generally, there should be no more than 3 such required experiments or major modifications for a "Major Revision" recommendation. If more than 3 experiments are necessary to validate the study conclusions, then you are encouraged to recommend "Reject".

Reviewer #1: (No Response)

**********

**Part III – Minor Issues: Editorial and Data Presentation Modifications**

Please use this section for editorial suggestions as well as relatively minor modifications of existing data that would enhance clarity.

Reviewer #1: (No Response)

**********

PLOS authors have the option to publish the peer review history of their article (what does this mean?). If published, this will include your full peer review and any attached files.

If you choose “no”, your identity will remain anonymous but your review may still be made public.

**Do you want your identity to be public for this peer review?**  For information about this choice, including consent withdrawal, please see our Privacy Policy.

Reviewer #1: No

# Editor report: Acceptance letter

**DOI:** 10.1371/journal.ppat.1014545.r004

**Authors:** Alexander Gorbalenya, Nuria Verdaguer

Dear Prof. Sun,

We are delighted to inform you that your manuscript, "Receptor-induced activation of MERS-CoV spike revealed by time-course cryo-EM.," has been formally accepted for publication in PLOS Pathogens.

We have now passed your article onto the PLOS Production Department who will complete the rest of the pre-publication process. All authors will receive a confirmation email upon publication.

Thank you again for supporting open-access publishing; we are looking forward to publishing your work in PLOS Pathogens.

Best regards,

Dominique Soldati-Favre

Editor-in-Chief

PLOS Pathogens
